# Isobutyrylcarnitine as a Biomarker of OCT1 Activity and Interspecies Differences in its Membrane Transport

**DOI:** 10.3389/fphar.2021.674559

**Published:** 2021-05-10

**Authors:** Ole Jensen, Johannes Matthaei, Henry G. Klemp, Marleen J. Meyer, Jürgen Brockmöller, Mladen V. Tzvetkov

**Affiliations:** ^1^ Institute of Clinical Pharmacology, University Medical Center Göttingen, Göttingen, Germany; ^2^ Institute of Pediatrics and Adolescent Medicine, University Medical Center Göttingen, Göttingen, Germany; ^3^ Institute of Pharmacology, Center of Drug Absorption and Transport (C_DAT), University Medicine Greifswald, Greifswald, Germany

**Keywords:** OCT1, SLC22A1 (OCT1), isobutyrylcarnitine, efflux, organic cation transporter, carnitine, Biomarker

## Abstract

Genome-wide association studies have identified an association between isobutyrylcarnitine (IBC) and organic cation transporter 1 (OCT1) genotypes. Higher IBC blood concentrations in humans with active OCT1 genotypes and experimental studies with mouse OCT1 suggested an OCT1-mediated efflux of IBC. In this study, we wanted to confirm the suggested use of IBC as an endogenous biomarker of OCT1 activity and contribute to a better understanding of the mechanisms behind the association between blood concentrations of carnitine derivatives and OCT1 genotype. Blood and urine IBC concentrations were quantified in healthy volunteers regarding intra- and interindividual variation and correlation with OCT1 genotype and with pharmacokinetics of known OCT1 substrates. Furthermore, IBC formation and transport were studied in cell lines overexpressing OCT1 and its naturally occurring variants. Carriers of high-activity OCT1 genotypes had about 3-fold higher IBC blood concentrations and 2-fold higher amounts of IBC excreted in urine compared to deficient OCT1. This was likely due to OCT1 function, as indicated by the fact that IBC correlated with the pharmacokinetics of known OCT1 substrates, like fenoterol, and blood IBC concentrations declined with a 1 h time delay following peak concentrations of the OCT1 substrate sumatriptan. Thus, IBC is a suitable endogenous biomarker reflecting both, human OCT1 (hOCT1) genotype and activity. While murine OCT1 (mOCT1) was an efflux transporter of IBC, hOCT1 exhibited no IBC efflux activity. Inhibition experiments confirmed this data showing that IBC and other acylcarnitines, like butyrylcarnitine, 2-methylbutyrylcarnitine, and hexanoylcarnitine, showed reduced efflux upon inhibition of mOCT1 but not of hOCT1. IBC and other carnitine derivatives are endogenous biomarkers of hOCT1 genotype and phenotype. However, in contrast to mice, the mechanisms underlying the IBC-OCT1 correlation in humans is apparently not directly the OCT1-mediated efflux of IBC. A plausible explanation could be that hOCT1 mediates cellular concentrations of specific regulators or co-substrates in lipid and energy metabolism, which is supported by our *in vitro* finding that at baseline intracellular IBC concentration is about 6-fold lower alone by OCT1 overexpression.

## Introduction

The organic cation transporter 1 (OCT1) is strongly expressed in human hepatocytes (Nies et al., 2009) and accelerates membrane transport of numerous endogenous metabolites, drugs and toxins (Nies et al., 2011; Koepsell, 2013). The *SLC22A1* gene, coding for human OCT1 (hOCT1), is genetically highly variable (Kerb et al., 2002; Shu et al., 2003; Seitz et al., 2015). In the European population, five common loss-of-function polymorphisms are known, which have significant consequences for the pharmacokinetics of drugs, such as fenoterol (Tzvetkov et al., 2018), metformin, and sumatriptan (Matthaei et al., 2016). Thiamine (vitamin B1) was identified as natural substrate of murine OCT1 (mOCT1) and hOCT1 (Chen et al., 2014) but thiamine pharmacokinetics are not dependent on hOCT1 genotype (Jensen et al., 2020).

**FIGURE 1:**
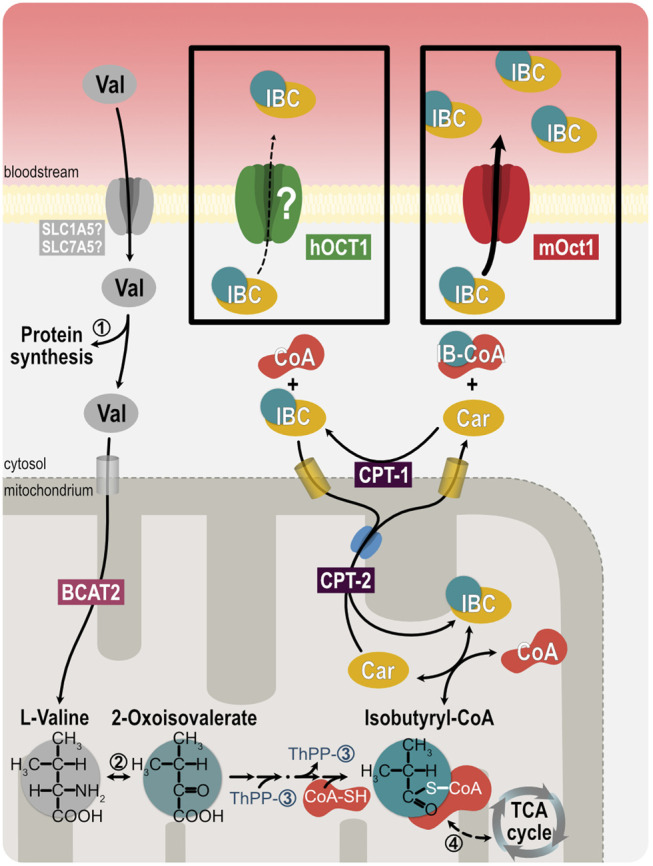
Illustration of isobutyrylcarnitine metabolism. Oxidation of branched-chain amino acids, such as valine, after uptake into mitochondria by SLC25A44 and the branched-chain amino acid transaminase 2 (BCAT2), lead to acylcarnitine intermediates, such as isobutyrylcarnitine. Exchange of carnitine and acylcarnitines across mitochondrial membranes exports the acyl residues into the cytosol. ➀ valyl tRNA ligase, ➁ branched-chain amino acid transaminase, ➂ 3-methyl-oxo-butanoate dehydrogenase, ➃ acyl-CoA dehydrogenase, CPT-1 carnitine-palmitoyltransferase 1, CPT-2 carnitine-palmitoyltransferase 2.

There is significant interest in the discovery of endogenous biomarkers reflecting the *in vivo* activity of drug metabolizing enzymes and the *in vivo* activity of drug membrane transport (Yee et al., 2016; Chu et al., 2017; Chu et al., 2018). For instance, human blood concentrations of N-methyl-nicotinamide, *N*-methyladenosine, glycochenodeoxycholate sulfate, and 6-beta-hydroxycortisol may reflect the *in vivo* activity of MATE, OCT2, OATP1B1, and OAT3, respectively. In genome-wide association studies, isobutyrylcarnitine (IBC) was strongly associated with *OCT1* genetic polymorphisms (Suhre et al., 2011) and may thus be a suitable endogenous biomarker of OCT1 activity (Luo et al., 2020). IBC is a metabolite of valine, when its acyl residue is transferred from isobutyryl-CoA to carnitine (Ramsay et al., 2001) (Figure 1). Acylcarnitines, in general, are amino acid or fatty acid breakdown products. Conversion of acyl-CoA to the acylcarnitine ester *via* carnitine acyltransferase is essential to maintain the pool of free coenzyme A (Ramsay et al., 2001). Because of the role of carnitine conjugation in buffering excessive fatty acids, acylcarnitine species are biomarkers of congenital metabolic diseases with disruption in peroxisomal or mitochondrial oxidation processes (Pedersen et al., 2006; Giesbertz et al., 2015).

The mechanisms of membrane transport of carnitine derivatives are controversial. The zwitterionic carnitine itself is transported by the almost ubiquitously expressed organic cation transporters OCTN1 and OCTN2 (Tamai et al., 1998; Ramsay et al., 2001; Koepsell, 2013; Salomon et al., 2019). Recently, an efflux function of OCT1 for IBC was proposed (Kim et al., 2017) based on transport experiments with mOCT1 and the relationship between human plasma carnitine derivatives and human OCT1 genotype was explained by that finding with murine OCT1. However, that explanation did not consider known major species differences in substrate selectivity and transport kinetics between mOCT1 and hOCT1, which are well-known and quite extensive (Gorboulev et al., 1997; Zhang et al., 1997; Green et al., 1999; Schmitt et al., 2003; Meyer et al., 2020). Short-chain acylcarnitine species are very hydrophilic with negative logD_7.4_ values ranging from −6.80 to −1.64 (Supplementary Table S1), indicating the necessity for transporter-mediated cell membrane passage. However, experimental evidence for IBC transport *via* organic cation transporters currently exists only for mOCT1.

With the studies presented here, we wanted to assess the suitability of IBC as endogenous biomarker of OCT1 genotype and phenotype. But most importantly, we wanted to elucidate the mechanisms behind the association between hOCT1 genotype and blood concentrations of IBC and other carnitine derivatives. Thus far, transport of IBC had only been studied with murine OCT1 but not with human OCT1 and efflux transport had been incompletely characterized. While performing these experiments, we soon discovered that IBC is not transported *via* human OCT1, neither into the cell nor out of the cell. Therefore, we performed several additional experiments to elucidate the mechanisms behind the association between OCT1 genotype and IBC blood concentrations. In this context we hypothesized that other OCT1-dependent endogenous substrates might regulate carnitine metabolism. Therefore, we studied the uptake of substances from human plasma into OCT1 active and OCT1 deficient cells by untargeted and semi-targeted metabolomics.

## Materials and Methods

### Clinical Study

In 65 healthy male and female individuals, IBC was measured in plasma after overnight fasting. Blood was taken in mornings on up to eight occasions with intervals of at least 1 week to compare intra- vs. interindividual variation and relation to OCT1 genotypes. Urine samples and corresponding plasma samples at the beginning and at the end of the urine collection period were collected by another 30 unrelated healthy volunteers after overnight fasting (3 h collection period). Renal clearance of IBC was calculated as the ratio of the amount of IBC excreted within the 3 h collection period over the plasma area under the concentration time curve of IBC from the same 3 h interval. Ethylenediaminetetraacetic acid was used as the anticoagulant for blood sampling in both studies. Plasma samples from volunteers that had participated in studies on the effect of OCT1 genotype on pharmacokinetics of fenoterol (Tzvetkov et al., 2018), sumatriptan (Matthaei et al., 2016), and proguanil (Matthaei et al., 2019) were used to correlate IBC blood concentrations with pharmacokinetics of these drugs. The studies were approved by the ethics committee of the University Medicine Göttingen and the relevant regulatory authories (EudraCT 2012-003546-33) and all volunteers had given written informed consent.

### Organic Cation Transporter 1 Genotyping

OCT1 genotyping was performed on DNA extracted from blood samples by solid-phase extraction. The genotyping procedure was described detailed elsewhere (Matthaei et al., 2016). In brief, primer extension assays were performed for the variants Ser14Phe (rs34447885), Arg61Cys (rs12208357), Cys88Arg (rs55918055), Pro117Leu (rs200684404), Ser189Leu (rs34104736), Gly401Ser (rs34130495), Met420del (rs202220802), and Gly465Arg (rs34059508). Almost all study samples were genotyped in duplicate, with 100% concordant results.

### Isobutyrylcarnitine Blood and Urine Concentration Analyses

Quantification of IBC plasma and urine concentrations was performed *via* liquid chromatography-coupled tandem mass spectrometry (LC-MS/MS) after precipitation. The detailed protocol is provided in the supplementary methods.

### Uptake and Efflux of Carnitine, Acylcarnitines, Valine, or Known Substrates

Transport experiments were performed with primary human hepatocytes (Thermo Fisher Scientific, Darmstadt, Germany) or HEK293 cells stably transfected to overexpress hOCT1 or mOCT1. As a control, cells transfected with the empty vector pcDNA5 were used. The generation and validation of the cell lines was described previously (Saadatmand et al., 2012; Meyer et al., 2020). Uptake or efflux of carnitine, acylcarnitines, or valine were performed with radiolabeled or deuterated substrates and quantified by scintillation counting or LC-MS/MS. For the latter, specific mass transitions and voltages were used (Supplementary Table S1). The cell number of each experiment was normalized by total protein measurement in representative wells by using the bicinchoninic acid assay (Smith et al., 1985). Detailed descriptions of the methods are provided in the supplementary files.

### Metabolomics

To identify endogenous substrates of mouse and human OCT1, we performed untargeted metabolomics. Plated HEK293 cells overexpressing mOCT1, hOCT1 or the empty vector were incubated with pooled fresh frozen plasma. After lysis and protein quantification for normalization purposes, lipids and proteins were removed by a modified Bligh and Dyer method (Bligh and Dyer, 1959). The detailed protocol of sample workup is provided in the Supporting Information section online. Detection of metabolites was performed by mass spectrometry on a *Xevo G2-S QToF*. Analysis was performed using *MassLynx 4.1* (Waters, Milford, United States), *Progenesis QI 2.4* (Nonlinear Dynamics, Newcastle upon Tyne, United Kingdom) as well as *Metaboanalyst 4.0* (Chong et al., 2019). Identification of metabolites was achieved by an in-house database as well as the HMDB database *via Progenesis* software (Wishart et al., 2007).

### Statistics

Linear regression was used to determine correlation between IBC plasma concentrations and known OCT1 substrates or metabolites. Statistical significance of differences between two groups was analyzed using the Student’s *t*-test and presentation of means and standard errors of mean (SEM). Comparisons between more than two groups were analyzed by one-way ANOVA with Tukey post-hoc test. *p < 0.05; *p < 0.01; ***p < 0.001. Trends of mean IBC plasma concentrations were analyzed by linear regression analysis. Unless noted otherwise, all *in vitro* analyses were performed at least with 3 independent replicates. The entire study sample available was used to compare IBC concentrations between carriers or two, one or zero fully active OCT1 alleles.

## Results

### Clinical Studies Confirmed Association of Plasma Isobutyrylcarnitine and Organic Cation Transporter 1 Activity

**FIGURE 2:**
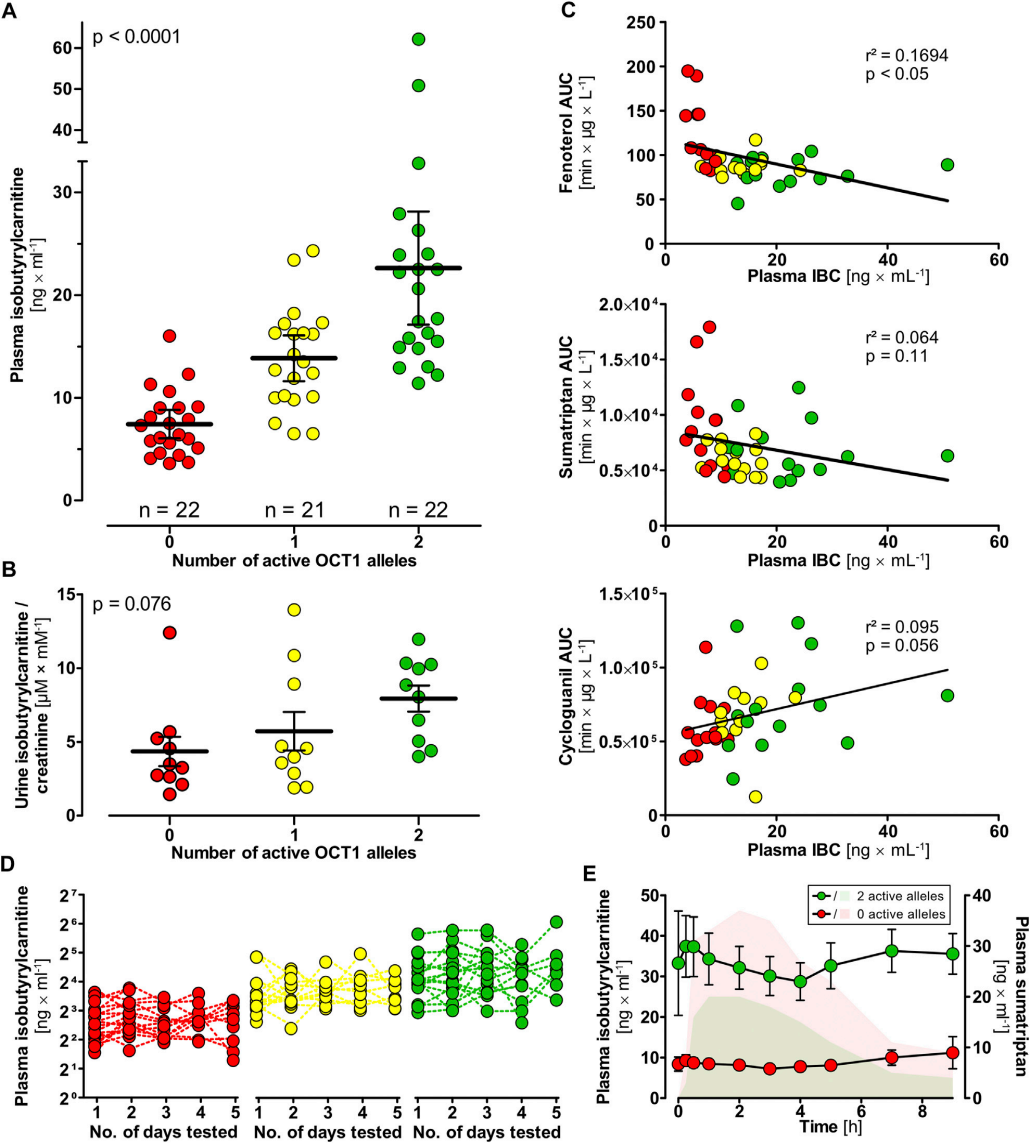
Isobutyrylcarnitine plasma concentrations in healthy volunteers (red circles, 0 active OCT1 alleles; yellow circles, 1 active OCT1 allele; green circles, 2 active OCT1 alleles). **(A)** Higher IBC plasma concentrations could be observed in individuals with active OCT1 compared to OCT1-deficient individuals (statistical testing performed using one-way ANOVA). **(B)** IBC urine concentrations were higher in individuals with active OCT1 compared with deficient OCT1 as well, however, not statistically significant (*p* = 0.076, linear regression analysis). **(C)** Mean IBC plasma concentrations showed genotype-dependent correlation with AUCs of the known OCT1 substrates fenoterol, sumatriptan, and cycloguanil (linear regression statistics). **(D)** High interindividual but low intraindividual variation in of plasma IBC was found supporting a strong genetic background for human plasma IBC. **(E)** IBC plasma concentrations (circles) after a single oral dose of 50 mg sumatriptan. Sumatriptan plasma concentrations of individuals with two reference alleles (green AUC) and individuals with zero active alleles are indicated. This data indicates that the IBC-OCT1 correlation is not only mediated by OCT1 genotype but also be OCT1 activity. Data are represented as the mean ± SEM.

To confirm the association between low plasma IBC and loss-of-function polymorphisms in OCT1, we analyzed the plasma IBC concentrations in 65 healthy volunteers. Plasma IBC concentrations were significantly higher in individuals carrying two wild-type alleles [22.6 ± 2.6 ng/ml (mean ± SEM)], compared to carriers of one (13.8 ± 1.1 ng/ml) or zero fully active OCT1 alleles (7.4 ± 0.7 ng/ml, *p* < 0.0001 in linear regression analysis, Figure 2A). It is to note, that the genotypes of the groups with one or zero active alleles are more diverse and comprise the variants OCT1*2 to OCT1*6 as inactive alleles (Supplementary Table S2). The measurements over time showed a stable course in each group classified by genotype (Figure 2D). Standard deviation between the means of all subjects was 8.73 ng/ml compared with a much lower standard deviation within all subjects of 4.24 ng/ml. The corresponding genetic component was 0.76, indicating that as much as 76% of the variation in IBC blood concentrations may be due to heritable factors (Kalow et al., 1998; Kalow et al., 1999). Amongst all tested factors possibly affecting the IBC blood concentrations, the OCT1 genotype was the most important one (multiple regression: β = 0.60, *p* = 3.9 × 10^−7^, r = 0.64). Other factors, such as age, sex, weight or body height were not significant.

Isobutyrylcarnitine concentrations in urine were highest in individuals carrying two reference alleles, followed by individuals with one and zero active alleles (not significant in one-way ANOVA, Figure 2B). Unlike in blood, the other acylcarnitines (acetylcarnitine, propionylcarnitine, 2-methyl-butyrylcarnitine, succinylcarnitine) were statistically not significantly associated with OCT1 genotype in urine. Mean renal clearances were 139 ml × min^−1^, 104 ml × min^−1^, and 234 ml × min^−1^ with an SEM of 10, 15, and 45 for volunteers with two, one and zero wild-type active OCT1 alleles (*p* < 0.01, linear regression analysis).

Plasma IBC concentrations correlated with the pharmacokinetics of the known OCT1 substrates fenoterol (*r* ^2^ = 0.169, *p* < 0.05) and sumatriptan (*r* ^2^ = 0.064, *p* = 0.11) as well as with cycloguanil, the metabolite of the OCT1 substrate proguanil (*r* ^2^ = 0.095, *p* = 0.056; Figure 2C). In healthy volunteers who had received a single oral dose of 50 mg sumatriptan (Matthaei et al., 2016), we observed a reduction of IBC occurring slightly delayed after the peak drug concentrations, but in volunteers with two active OCT1 alleles only (Figure 2E).

### Mechanisms Underlying the Isobutyrylcarnitine Organic Cation Transporter 1-Genotype Association

**FIGURE 3:**
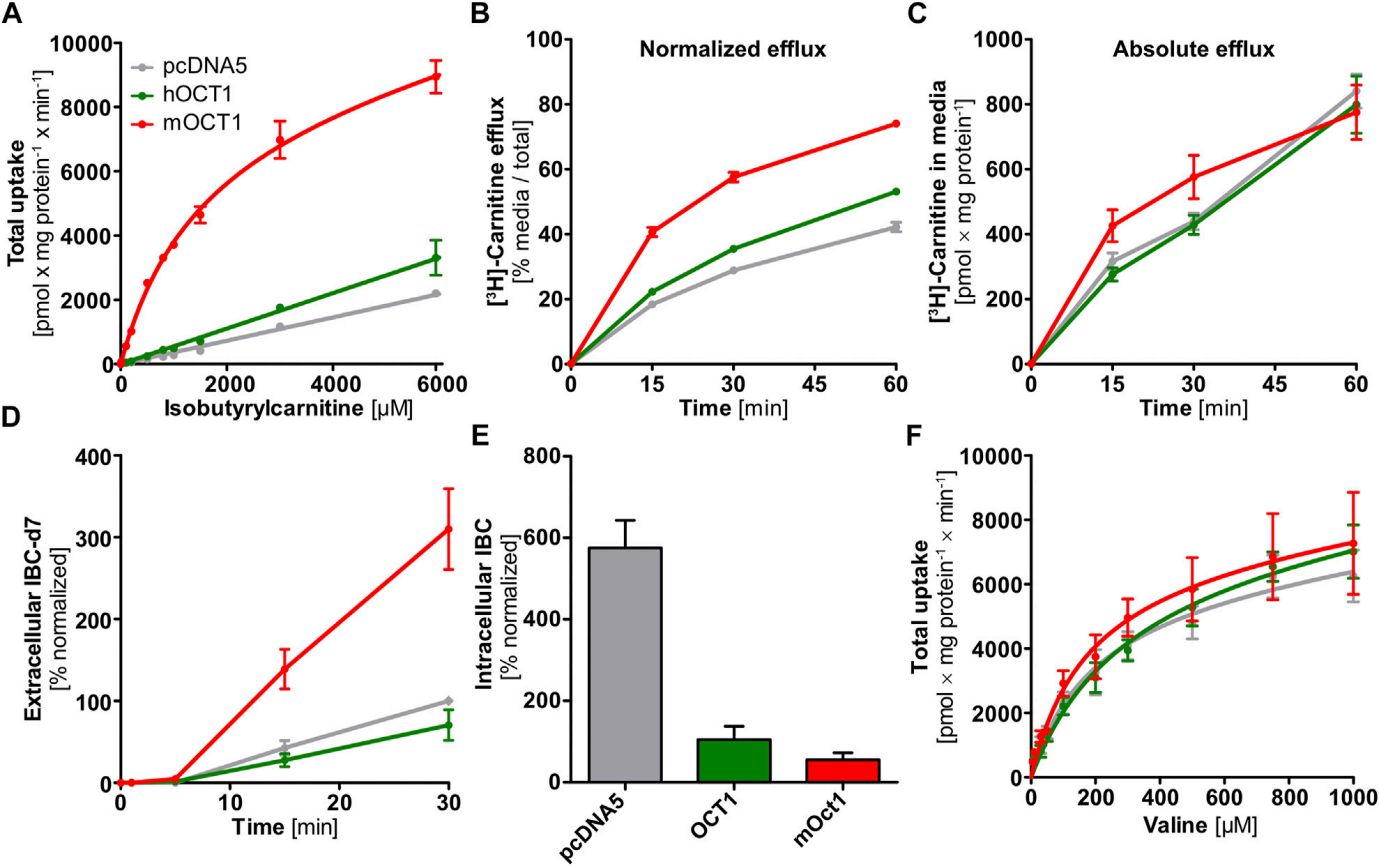
*In vitro* uptake and efflux experiments with carnitine, isobutyrylcarnitine, or valine. **(A)** In contrast to HEK293 cells overexpressing mOCT1, hOCT1 did not show uptake of IBC significantly above the uptake seen in empty-vector control cells (pcDNA5). **(B)** HEK293 cells overexpressing mOCT1 showed increased efflux of radiolabeled carnitine, compared to hOCT1 and empty vector control (pcDNA5) when calculated in relation to total carnitine in medium and cells. **(C)** Absolute carnitine efflux did not differ between cells overexpressing hOCT1 and empty vector control and also with mOCT1 there was a moderate effect only. **(D)** Efflux of IBC-d7 was observed after incubation with valine-d8 only for mOCT1. **(E)** Intracellular IBC concentrations under regular culturing conditions depended strongly on the overexpression of OCT1 and are higher without hOCT1 or mOCT1 (*p* < 0.0001, one-way ANOVA with Tukey post-hoc test). **(F)** Valine was studied as the precursor of IBC, Valine uptake by mouse and human OCT1 compared to empty-vector control cell line (pcDNA5). **(A–F)** Data are represented as the mean ± SEM from at least three independent experiments. Total uptake implies the intracellularly accumulated substance normalized by total protein and time, without subtraction of uptake into empty vector-overexpressing cell line “pcDNA5”.

First, we wanted to characterize transport kinetics of IBC with human OCT1 (hOCT1). In HEK293 cells overexpressing hOCT1, uptake of IBC did not show saturated transport characteristics (Figure 3A). Moreover, uptake of IBC in these cells could not be inhibited by addition of the well-established OCT1 inhibitors 1-methyl-4-phenylpyridinium (MPP+) or 4-[4-(dimethylamino)styryl]-N-methylpyridinium (ASP+, Supplementary Figure S1). Human OCT1 is therefore unlikely to be a mediator of IBC cell uptake. In contrast, mOCT1 showed low affinity-high capacity influx transport with a K_M_ value of 1.47 ± 0.17 mM (mean ± SEM) and a v_max_ of 8.50 ± 0.41 nmol × mg protein^−1^ × min^−1^. The hOCT1 and mOCT1 cell lines were validated with numerous substrates (Matthaei et al., 2016; Tzvetkov et al., 2018; Matthaei et al., 2019) with an excellent correlation between *in vitro* intrinsic clearance and *in vivo* pharmacokinetic data, indicating that this model cell line is reflecting membrane transport in humans quite well.

The corresponding transport experiments with the human carnitine transporter hOCTN2 showed a K_M_ of 72.7 ± 18.6 µM (mean ± SEM) and a v_max_ of 2.06 ± 0.08 nmol × mg protein^−1^ × min^−1^ for IBC, which underlines the well-known capabilities of hOCTN2 to transport not only carnitine but also acylcarnitine species in humans. Altogether, in contrast to mOCT1, hOCT1 was not capable of accelerating cell uptake of IBC, but IBC may be transported by hOCTN2.

### Substantial Interspecies Differences in Carnitine Efflux Transport

The IBC-OCT1 genotype association might also be explained by an effect of OCT1 genotype on hepatocellular availability of the precursors carnitine and valine. However, the efflux of carnitine was strongly increased upon overexpression of mOCT1 but not, or only to a very moderate extent, by hOCT1 (Figure 3B) when normalized by total carnitine after the incubation phase. Compared to reference hOCT1, cell lines expressing common loss-of-function hOCT1 allelic variants (hOCT1*2 and *3) showed even reduced carnitine efflux and ranged between reference hOCT1 and the empty-vector control cell line (Supplementary Figures S2A,B). However, absolute [^3^H]-carnitine efflux into the cell culture medium was not significantly increased by overexpression of mOCT1 or hOCT1 (Figure 3C), which indicates that seemingly existing differences in carnitine efflux occurred solely by unequal preloading. Preloading was much higher with mOCT1 compared with hOCT1. This shows that simple normalization after unequal preloading is not sufficient and conveys a misinterpreting message compared to efflux with similar intracellular starting concentrations. However, the capacity of hOCT1 to catalyze carnitine influx was shown earlier (Kim et al., 2017) and confirmed by the higher preloading in hOCT1 active cells compared with control cells. Thus, one explanation why IBC was higher with high OCT1 activity might simply be a better supply of carnitine in hepatocytes.

**FIGURE 4:**
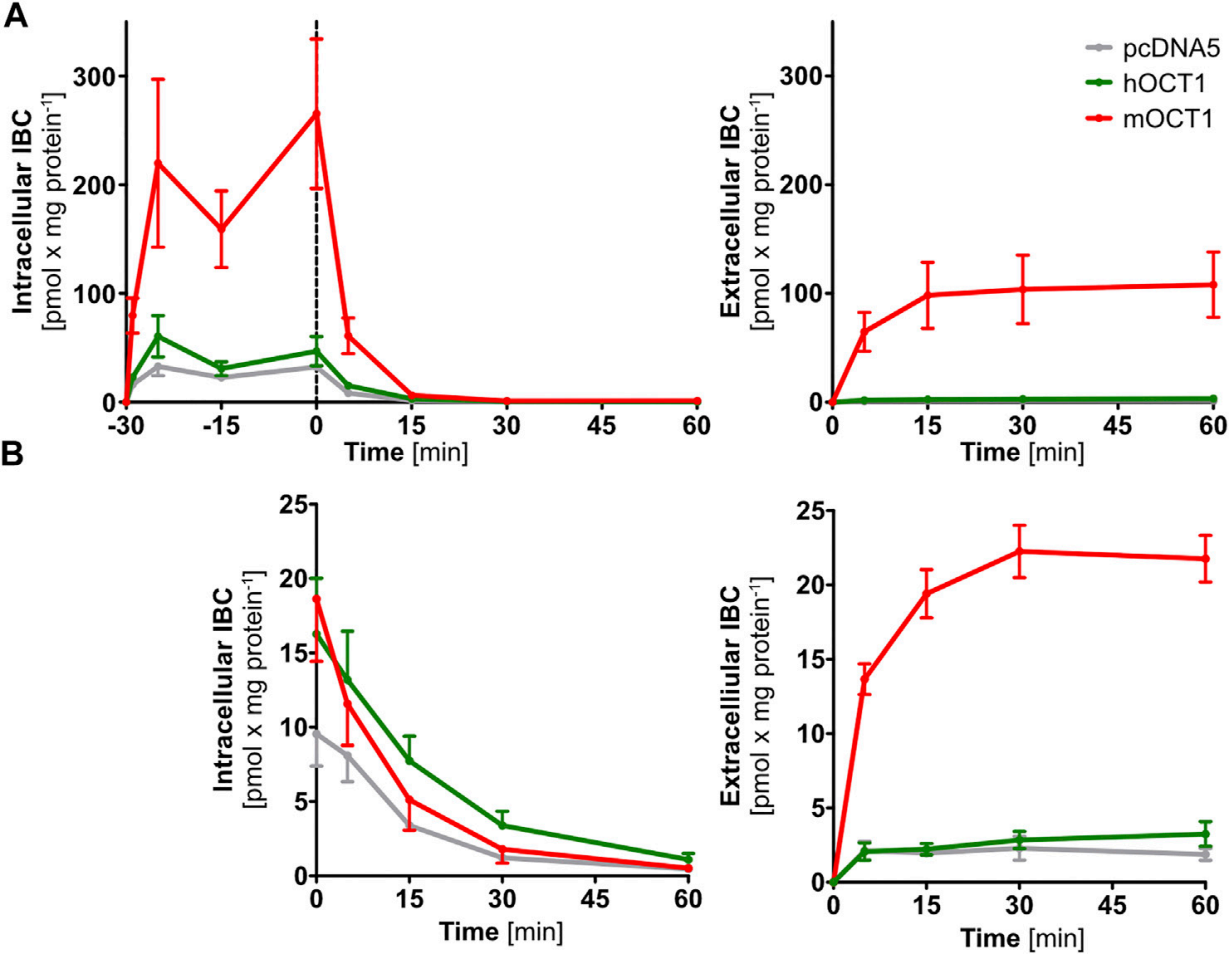
Time-dependent isobutyrylcarnitine efflux. Absolute efflux of IBC without **(A)** and with **(B)** adjustment of preloading IBC concentrations revealed IBC efflux capabilities of mOCT1 but not hOCT1. In the upper right part, intracellular concentrations are shown during the course of preloading and then, after stop of preloading by changing the media, the efflux is shown. In the other three figures, only the efflux phase is shown. Data are represented as the mean ± SEM and result from at least three independent experiments.

To provide direct evidence for mOCT1-and hOCT1-mediated IBC efflux transport, we investigated the efflux of IBC after preloading with IBC. Because substance efflux is always the sum of (linearly concentration dependent) intracellular concentration of the substance undergoing efflux plus the transporter mediated acceleration of efflux, normalization of the starting condition is very important to characterize transporter-mediated efflux. Normalized efflux was high in mOCT1 overexpressing cells, limited only by intracellular amounts (Figure 4A). After 30 min, almost all preloaded IBC was effluxed into the extracellular medium. In contrast, mOCT1 did apparently not accelerate IBC efflux. As illustrated, there was only a minor difference between hOCT1 and the empty-vector control cell line. However, much higher intracellular IBC concentrations could be found after the 30 min preloading period in mOCT1 overexpressing cells, which reflects the differences in IBC uptake between mOct1 and hOCT1 (Figure 4A). As a consequence, absolute IBC efflux was dramatically stronger *via* mOCT1, compared to human OCT1 and negative control. Thus, the observed efflux activity published earlier (Kim et al., 2017) and found in our experiments normalized by total [^3^H]-carnitine (Figure 3B) might be the result of the increased preloading prior to measurement of efflux.

To differentiate whether the observed efflux by mOCT1 was the result of higher preload or a really higher efflux activity, IBC concentrations for pre-incubation were adjusted to match the concentration in empty-vector control cells after the preloading phase. After adjustment, not any efflux of IBC was found by hOCT1, neither with nor without normalization to total IBC. In contrast, mOCT1 overexpressing cells showed an increased efflux into the medium, especially in absolute terms (Figure 4B; Supplementary Figure S3B).

### Formation and Efflux of d9-Acylcarnitines

**FIGURE 5:**
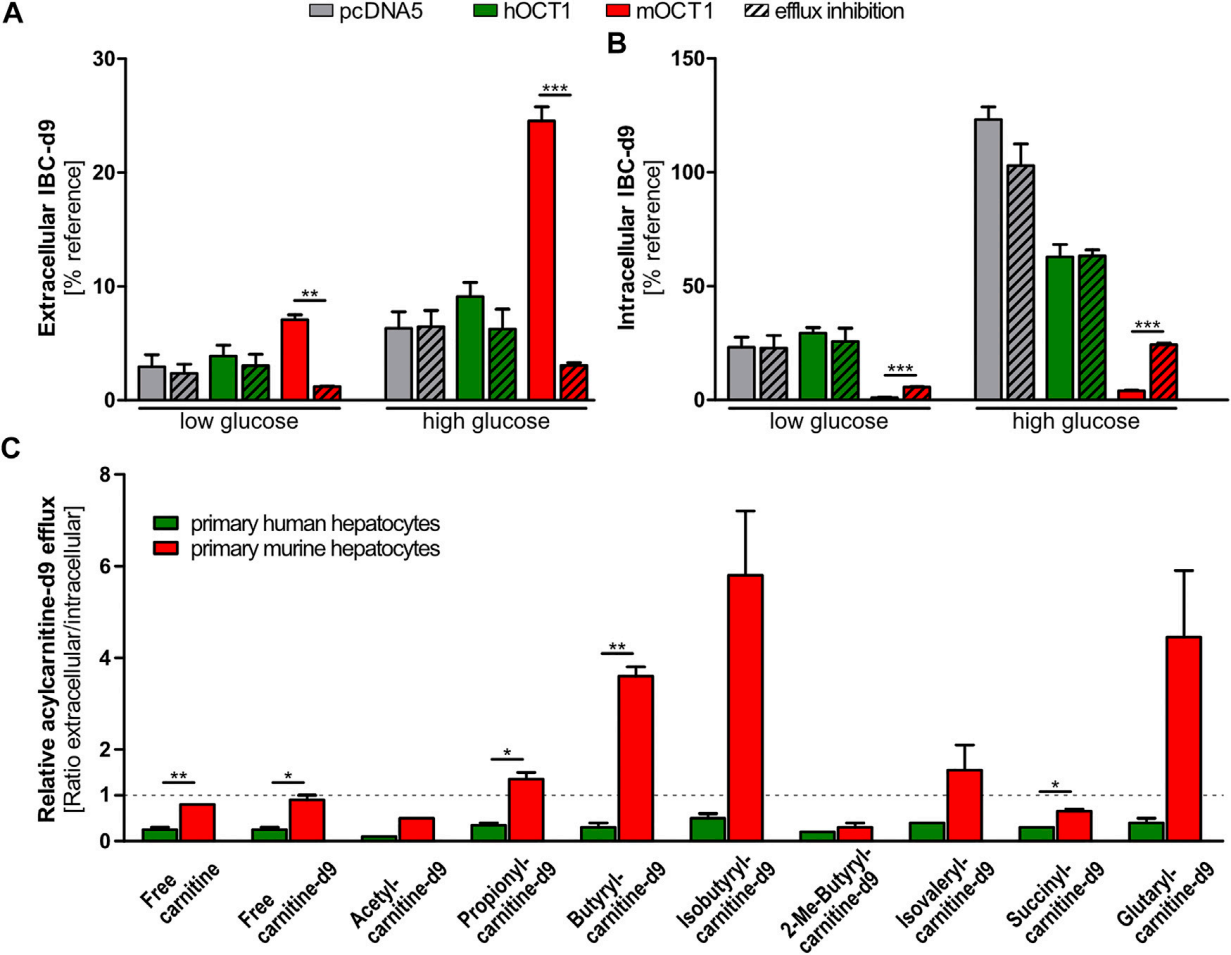
Efflux of acylcarnitines. **(A)** Efflux of acylcarnitines after incubation with carnitine-d9 led to increased extracellular and reduced intracellular isobytyrylcarnitine and other acylcarnitine species. Inhibition of OCT1 by desipramine (indicated with ‘+‘) reduced or hindered this efflux. **(B)** Murine and human primary hepatocytes revealed distinguishable patterns of intra- and extracellular acylcarnitine species after preloading with deuterated carnitine (carnitine-d9). Again, only experiments with mouse OCT1 showed a consistent efflux activity of mOCT1 (red columns). Glucose concentration in the medium may change metabolic pathways but as seen, the relative effects of hOCT1 and mOCT1 were the same irrespective of glucose supply. **(A)** and **(B)** Data are represented as the mean ± SEM from at least three independent experiments. Normalization was conducted by comparison to intracellular carnitine-d9 post efflux. **(C)** Efflux from primary murine or human hepatocytes appears different for several acylcarnitine species. Measurement in supernatant medium and in cells showed increased efflux by murine hepatocytes for most investigated acylcarnitines. Data are represented as the mean ± SEM. Statistical analysis of two groups were performed using the Student’s *t*-test,*p < 0.05; *p < 0.01; ***p < 0.001.

OCT1-dependent differences in plasma concentrations of IBC and other acylcarnitines could depend on their biosynthesis. To track the intracellular formation and subsequent efflux of acylcarnitine species, cells overexpressing mouse or human OCT1 were incubated with deuterated carnitine (carnitine-d9). By this, also the produced acylcarnitines were deuterated and could be specifically quantified (Supplementary Table S1) showing that human OCT1 overexpressing cells did not differ from empty-vector control cells with respect to efflux of acylcarnitines without or with inhibition of OCT1. In contrast, cells overexpressing mOCT1 revealed lower intracellular concentrations of IBC-d9, independent of glucose concentrations in the medium (Figure 5A and Supplementary Figure S4). Additional experiments with high and low glucose in the media were performed because catabolism of lipids and amino acids might significantly depend on the glucose supply. Correspondingly, the extracellular concentrations of these acylcarnitines were elevated compared to cells overexpressing hOCT1 or empty-vector control cells. These effects of mOCT1 could be reduced or even inverted by addition of the OCT1 inhibitor desipramine during the efflux phase of the experiment. Similar observations were also made for other acylcarnitine species, such as butyrylcarnitine-d9, hexanoylcarnitine-d9, and many more (Supplementary Figure S4). Interestingly, the concentration of glucose in the medium did significantly influence the amount of formed product. However, the effect of OCT1 was similar with high and low glucose (Figure 5A). Again, this experiment confirmed that only mOCT1, but not hOCT1 acts as efflux transporter of acylcarnitine derivatives.

### Comparative Efflux of Acylcarnitine Species by Murine and Human Primary Hepatocytes

In addition to cells overexpressing mouse or human OCT1, primary hepatocytes were used to evaluate the capability of both species to facilitate efflux of acylcarnitines. Results showed that—in those cases in which the concentrations differed significantly—remaining intracellular amounts of acylcarnitine species were lower in mouse hepatocytes and higher in human hepatocytes after the 30 min efflux period (Supplementary Figure S5). Vice versa, extracellular amounts of acylcarnitine species were higher in the medium of mouse hepatocytes and lower in the medium of human hepatocytes, which resulted in increased extracellular/intracellular ratios (Figure 5C). The most striking results were found for butyrylcarnitine, of which extracellular concentrations were more than twice as high in the medium of mouse hepatocytes, while intracellular concentrations were about thrice as high in human hepatocytes. This confirmed the comparatively enhanced acylcarnitine efflux capability of mouse hepatocytes compared to human hepatocytes, as it was shown by stable transfected HEK293 cells overexpressing mouse or human OCT1.

### Tracing Organic Cation Transporter 1 Depending Isobutyrylcarnitine by Use of Deuterated Valine

Uptake of valine, the IBC precursor, was not different between the empty vector control and OCT1 overexpressing cells, which excludes an increased valine uptake as the direct cause for elevated IBC levels (Figure 3F). Saturation of uptake was similarly overserved for the empty-vector control cell line as well—probably mediated by amino acid transporters.

Use of valine-d8 offered another chance to trace the mOCT1 vs. hOCT1 dependent cellular fate of isobutyrylcarnitine. Conversion to isobutyryl-d7-CoA and the subsequent formation of IBC-d7 from valine-d8 was tracked based on the deuteration-altered mass. The intracellular accumulation of IBC-d7 was lower in mOCT1 and hOCT1 overexpressing cells compared to empty-vector control cells, while the extracellular culmination of IBC-d7 was the highest in mOCT1 overexpressing cells (Figure 3D). These findings indicate the involvement of mouse and human OCT1 in transport of substances influencing cell metabolism. However, in connection with the other experiments (Figures 3,4) this does neither proof IBC uptake nor efflux by hOCT1. It is to note that a general imbalance was seen in HEK293 cell lines under regular culturing conditions. Non-deuterated IBC was increased intracellularly in the control cell line by about ten-fold compared to the same cell line overexpressing mOCT1 and about five-fold compared to the cell line overexpressing hOCT1 (Figure 3E). This indicates a constant efflux of IBC or general differences in metabolism, leading to varying formation.

### Comparative Efflux of Other Known Substrates by Mouse and Human Organic Cation Transporter 1

To evaluate a potentially diverging capability of OCT1 to facilitate efflux in general, we investigated the efflux of five known OCT1 substrates. After adjusting intracellular preloading concentrations to those in the empty-vector control cell line, the efflux was characterized by quantifying the substances in the supernatant and the cell lysate. None of the tested substrates were subject to efflux by mOCT1 (Supplementary Figure S6) and only metformin efflux transport was enhanced by hOCT1 overexpression compared with the control cell line (Supplementary Figure S6D). Lower extracellular concentrations of proguanil in cells overexpressing hOCT1 compared to control cell line point towards the simultaneous uptake (Supplementary Figure S6B). Overall, the differences between mOCT1 and hOCT1 concerning IBC efflux could not be observed for other OCT1 substrates. In particular, these experiments with 5 drugs may indicate that there is no generally higher propensity of mOCT1 to catalyze efflux transport compared with hOCT1.

### Metabolomics Mouse and Human Organic Cation Transporter 1 Uptake Profile

**FIGURE 6:**
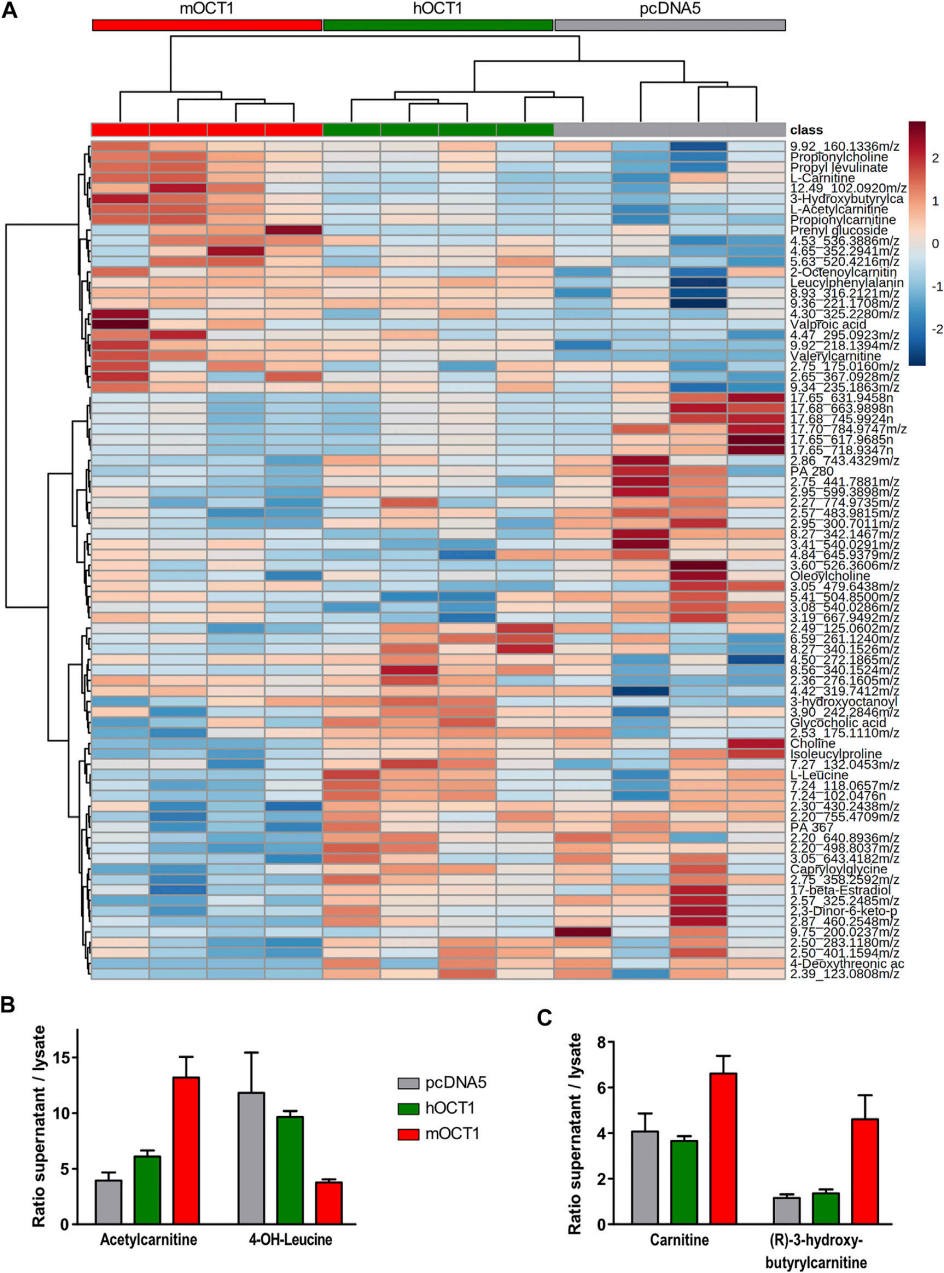
Metabolomic analyses from hOCT1 or mOCT1 overexpressing HEK293 cells **(A)** Hydrophilic interaction chromatography coupled with positive ionization mode mass spectrometry led to identification of metabolites differentially taken up from human normal donor blood plasma. If substances could not unambiguously be identified based on their masses, the retention times and the mass-to-charge ratios are provided. Data for heatmap was normalized and clustering of the 80 features with lowest ANOVA *p*-value is shown. Euclidean distance and Ward-clustering were used. As indicated by the red, green and gray bar showing the mOCT1, hOCT1 and empty vector transfected cell lines, with one exception clustering reflected the different effects of mOCT1 and hOCT1 very well **(B)** Identification by retention time and mass revealed varying uptake by hOCT1 and mOCT1. Mean ± SEM, *n* = 4 **(C)** Differences were also observed for substances identified only by mass database. Data are represented as the mean ± SEM from four independent experiments.

The association between OCT1 genotype and blood IBC concentrations may be explained by a more complex metabolic crosstalk with involvement of other substances not yet known to us in the present context. Therefore, untargeted and semi-targeted metabolomics analyses were performed on human plasma and lysates of HEK293 cells overexpressing mouse or human OCT1 after incubation with human plasma. The extracellular/intracellular ratio was used to identify the top 80 metabolites, which were differentially transported into the cells (Figure 6A). Amongst the positively ionized metabolites, the most striking differences were observed for acetylcarnitine and 4-OH-L-leucine, for which mOCT1 overexpressing cells showed an increased (acetylcarnitine) or decreased (4-OH-leucine) extracellular-to-intracellular ratio compared to both, hOCT1 overexpressing and empty-vector control cells (Figure 6B). Also 3-hydroxybutyrylcarnitine (an intermediate in the catabolism of lysine and tryptophan) and L-carnitine were subject to mOCT1 efflux with a 3.5-fold and a 1.5-fold increase of the ratio, respectively (Figure 6C).

Also several anionic substances were affected by hOCT1 or mOCT1 overexpression. The extracellular-to-intracellular ratio for OCT1 was increased for 14-hydroxymyristic acid (14-hydroxytetradecanoic acid) by about 2.5-fold, compared to negative control and mOCT1. Furthermore, the ratio for 5-methyl cytidine, docosahexaenoic acid and 9-oxodecanoic acid was reduced by mOCT1 overexpression (data not shown).

**FIGURE 7:**
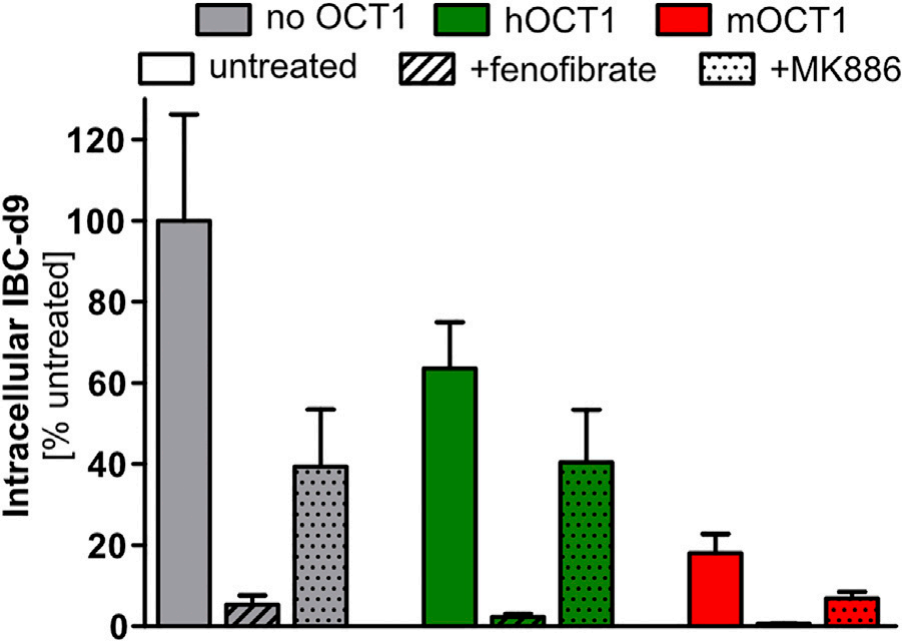
Isobutyrylcarnitine depletion inducible by PPARα activation. Pretreatment for 24 h with 33 µM fenofibrate, an activator of PPARα, led to strong reduction of intracellular IBC. But somehow unexpectedly, the PPARα inhibitor MK886 also reduced intracellular IBC. Data are represented as the mean ± SEM from four independent experiments. This data illustrates that the OCT1 effect on IBC concentrations may also be due to more complex signaling interactions and not due to an OCT1 mediated membrane transport of IBC.

Since our *in vitro* systems could not identify any relevant IBC transport by human OCT1, we speculated that the IBC-OCT1 correlation may be due to a more complex metabolic or signaling crosstalk. It might well be that OCT1 transports endogenous regulators of energy metabolism and the resulting differences might then result in the correlation between OCT1 activity and IBC. A strong regulator of energy metabolism is PPARα. Therefore, we studied the effects of the PPARα agonist fenofibrate and the antagonist MK886 on intracellular IBC concentrations. As seen in Figure 7, there was a remarkable effect of these transcriptional modulators on IBC, but the effect did not significantly differ depending on presence or absence of human OCT1.

## Discussion

Human organic cation transporter OCT1 is characterized by extensive mostly genetic variation with complete lack of activity in several percent of many populations on the one hand and very high expression and activity in the remaining population. Understanding the endogenous physiological implications of this variation is a most exciting issue of pharmacogenomics. This study confirmed that carriers of hOCT1 genotypes conferring reduced or deficient activity have on average more than threefold lower IBC blood concentrations than carriers of OCT1 reference genotype. IBC blood concentrations showed high interindividual but low intraindividual variation, indicating significant heritability of IBC formation and/or transport. IBC blood concentrations correlated with pharmacokinetics of known OCT1 substrates confirming suitability of IBC as an endogenous *in vivo* biomarker of OCT1 activity. With all the clinical and experimental data presented here we aimed to elucidate the mechanisms behind the relationship between human OCT1 activity and concentrations of carnitine derivatives in human blood. Experiments with cell lines overexpressing hOCT1 and mOCT1 and with more “naturalistic” primary human hepatocytes should contribute to the understanding of the mechanisms behind the OCT1 genotype-dependent differences in IBC concentrations (Figures 2,4,5) (Suhre et al., 2011; Kim et al., 2017). These experiments revealed that hOCT1, in contrast to mOCT1, did not exhibit any efflux activity for acylcarnitines. Experiments with primary hepatocytes from both species underlined these interspecies differences between hOCT1 and mOCT1, while other hepatic transporters might contribute in these primary cells as well. None of the results were compatible with the previously suggested explanation for the IBC-OCT1-genotype association, namely that hOCT1 would be an IBC efflux transporter (Kim et al., 2017). Apparently the simple experimental systems of HEK cells overexpressing hOCT1 or primary human hepatocytes expressing OCT1 (Figure 5B) cannot mimic or explain the human IBC OCT1-genotype association. Since all clinical data presented here are compatible with the hypothesis that OCT1 is an efflux transporter for IBC, the most obvious explanation is that OCT1 behaved in all our cell culture experiments differently than in the human body. While we cannot finally exclude this, we and other investigators have long lists of OCT1 substrates where the *in vitro* data correlated excellently with the human pharmacokinetic data (Shu et al., 2008; Tzvetkov et al., 2011; Fukuda et al., 2013; Tzvetkov et al., 2013; Matthaei et al., 2016; Tzvetkov et al., 2018). Based on that we still have to seek for other mechanisms behind the association between human blood plasma IBC concentrations and OCT1 genotype and OCT1 activity as well (Figure 2). However, the fact that known inhibitors had no effect on the uptake of IBC *in vitro* (Supplementary Figure S2) while the intake of sumatriptan led to a decrease in IBC plasma concentrations *in vivo* (Figure 2E) suggests that the connection between OCT1 activity and increased IBC plasma concentrations cannot simply be the result of inhibition of IBC transport by OCT1. Since OCT1 is also a carnitine transporter, inhibition of carnitine hepatocellular uptake might be an explanation why carnitine derivatives are lower when OCT1 activity is low. However, there is at least one other strong carnitine uptake transporter, OCTN2, and it is questionable why hOCT1 should be limiting in this situation.

**FIGURE 8:**
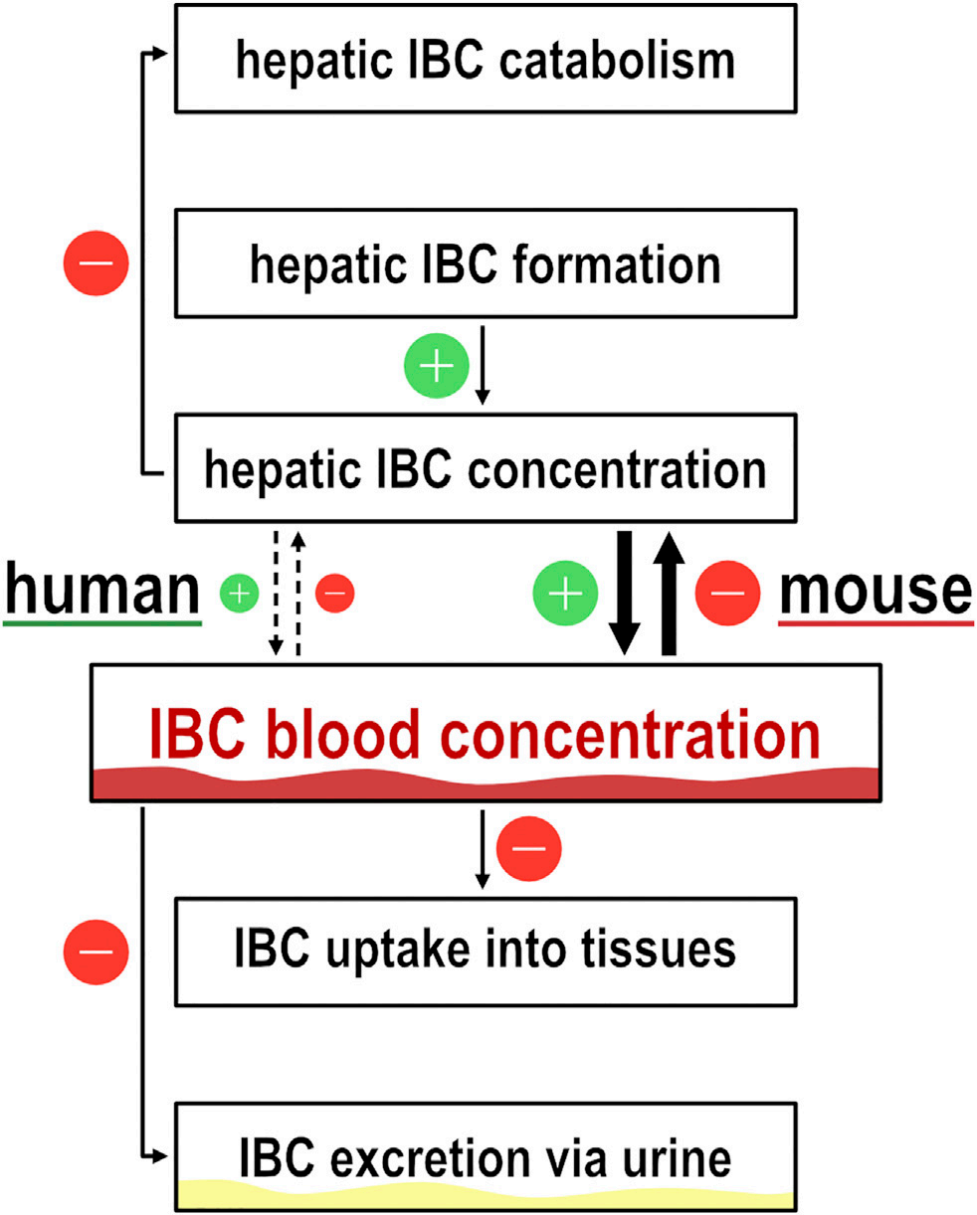
Illustration of isobutyrylcarnitine dynamics. Flow chart to illustrate the dynamics of isobutyrylcarnitine blood concentrations. Icons with ‘+’ and ‘−’ illustrate the effect on IBC blood concentration by the respective pathway.

Generally, high IBC blood concentrations, and high blood concentrations of other carnitine derivatives as well, may be explained by higher formation rate and/or by lower elimination rate (Figure 8). The first alternative, formation rate of IBC depending on expression of OCT1 may be higher if OCT1 mediates efflux or simply only intracellular binding of IBC and thus preventing degradation of IBC *via* the citric acid cycle. The second alternative, elimination of IBC, is mostly *via* renal glomerular filtration and tubular secretion processes (Rebouche and Paulson, 1986; Mancinelli et al., 1995). Expression of OCT1 in renal tubular cells is controversial. But if it is true that OCT1 is expressed at the apical side of renal tubular cells (Tzvetkov et al., 2009), high OCT1 activity could result in higher re-absorption and thus lower elimination rate. This could be observed in our measurements of IBC in urine (Figure 2B) and resulting renal clearances were indeed higher in carriers of two functionally inactive OCT1 alleles than in those with active OCT1. This would be compatible with tubular reabsorption of IBC. However, the extent of differences in IBC plasma concentration cannot be explained by a moderate renal reabsorption and this concept again would require OCT1 mediated influx transport which was not observed in our hOCT model cell lines.

High human IBC blood concentration was interpreted to be the result of high OCT1 efflux activity, but numerous other explanations may similarly explain these correlations. Generally, high concentrations of carnitine derivatives may reflect the status of cell energy metabolism (Roe et al., 1984; Roe et al., 1998; Sass et al., 2004). A relative thiamine deficiency might result in a shift from oxidative metabolism to fermentation, since several biochemical reactions within the citric acid cycle, but also the conversion of 2-oxoisovalerate to isobutyryl-CoA, depend on the cofactor thiamine pyrophosphate (Perham, 2000). Reduced activity of isobutyryl-CoA dehydrogenase leads to increased plasma levels of isobutyrylcarnitine (Koeberl et al., 2003). Similarly, elevated isovalerylcarnitine could be found as a result of impaired isovaleryl-CoA dehydrogenase activity (Roe et al., 1984). Thus, at a first glance thiamine might be the clue for the IBC-OCT1 relationship in humans. However, under thiamine deficiency, blood concentrations of acylcarnitine derivatives should be lower in OCT1 deficiency-coding than in active OCT1 genotypes. In addition, as shown by several clinical and cell culture experiments, OCT1 is not relevant for thiamine pharmacokinetics in humans and is only one of several thiamine transporters in human cell lines (Jensen et al., 2020).

Another interesting point is, whether or not OCT1 can function as an efflux transporter. Based on current mechanistic understanding of the alternating access model proposed for OCT1 (Koepsell, 2015), both influx and efflux transport may be mediated by this transporter but Hendrickx *et al.* identified only 3 out of 354 substances potentially transported out of the cell by hOCT1 (Hendrickx et al., 2013). Here we tested this in more detail for known OCT1 substrates, such as fenoterol, metformin, proguanil, ranitidine, and sumatriptan, and interestingly only for metformin an efflux transport activity was seen with hOCT1 (Supplementary Figure S6).

The experiments on ^3^H-carnitine efflux that led to the recently proposed efflux of IBC through hOCT1 as the explanation for elevated blood concentrations (Kim et al., 2017) were in general resembled in this work. Here, for the first time, the efflux of explicitly IBC revealed strong interspecies differences, with hOCT1 being much less capable of carnitine efflux than mOCT1, which stand in contradiction to the previous explanation. In addition, differences in uptake and intracellular preloading were considered and implied into *in vitro* transport experiments. These interspecies differences of OCT1 are well known and have been shown in multiple occasion, so that the transfer of findings across species cannot be performed unseen (Gorboulev et al., 1997; Zhang et al., 1997; Green et al., 1999; Schmitt et al., 2003; Meyer et al., 2020). Eventually, alternative explanations for the correlation of elevated plasma levels and an active OCT1 are needed.

The relationship between blood concentrations of IBC might be mediated by OCT1-dependent uptake of valine, the precursor of isobutyryl-CoA/IBC, by OCT1—surplus of valine might lead to a surplus of IBC, which gets exported. But this hypothesis was not supported by our experiments (Figure 3), there is no valine transport *via* OCT1. IBC formation might be regulated by other metabolites, therefore we searched for other metabolic differences using metabolomics analyses. Indeed, there were numerous differences between OCT1 expressing cells compared with empty-vector control cells (Figure 6; Supplementary Table S3).

The plasma acylcarnitine composition in general reflects the cellular acyl-CoA pattern (Costa et al., 1999) and fasting increases acylcarnitines at the expense of free carnitine (Frohlich et al., 1978). Therefore, the presence or absence of a functional OCT1 could induce a shift in cellular metabolism, which becomes manifest as discriminative plasma levels of isobutyrylcarnitine. The metabolomics data presented in this study provides a reasonable picture underpinning the differences between human and murine OCT1 in uptake and efflux of their substrates. Intracellular concentrations of probably more than 100 endogenous substances was different depending on hOCT1 or mOCT1 overexpression, but extensive further research is needed to disentangle which of these differences are physiologically relevant.

Upregulation of PPARα is known to lead to an increase of short-chain acylcarnitines in urine (Patterson et al., 2009). There are some striking similarities between these PPARα effects on carnitine derivatives described by Patterson et al., 2009, and the effects of OCT1 genotype in humans found here. Also, our finding from the untargeted metabolomics assay that some longer-chained fatty acid derivatives (substances very unlikely to be transported *via* OCT1) were different depending on OCT1 expression might be compatible with the hypothesis that concentrations of endogenous regulators of PPARα are modulated by OCT1 explaining the IBC-hOCT1 relationship and this might also explain the association between OCT1 and lipid metabolism (Liang et al., 2018). In contrast to previously reported increased short-chain acylcarnitines upon PPARα activation *in vivo* (Patterson et al., 2009), in our *in vitro* experiments PPARα activation led to a strong reduction of IBC, but independent of hOCT1/mOCT1 overexpression. The experiments with a PPARα agonist and an antagonist did, however, not support that the OCT1 effect on IBC is mediated by an endogenous regulator of PPARα. However, effect of PPARα modulators being much bigger than that of OCT1 (Figure 7) illustrates that generally a more complex metabolic or signaling crosstalk could be the basis of the OCT1/IBC association.

In conclusion, here we confirmed the previous findings (Suhre et al., 2011; Rodrigues et al., 2018) that plasma IBC is strongly associated with OCT1 activity (Figure 2). However, the physiological basis of the association remains unclear. In this study, we showed that the previous suggested explanation that OCT1 acts as a hepatic efflux transporter for IBC (Kim et al., 2017) is of only limited validity in humans. We showed that mouse OCT1 is both, an uptake and efflux transporter of IBC, but human OCT1 is neither an uptake transporter nor an efflux transporter of IBC. Once again, this example shows the difficulties in the translation from mouse models to human conditions, and simple explanations of human physiology based on data from mice may sometimes point the wrong way. The precise mechanisms leading to the association between OCT1 activity and plasma IBC in humans have to be elucidated in the future, but plasma IBC association with OCT1 levels have been confirmed in multiple studies and thus plasma IBC could be regarded a valid biomarker for OCT1 activity.

## Data Availability

The original contributions presented in the study are included in the article/Supplementary Material, further inquiries can be directed to the corresponding author.
