# Supplementary figures and images for: Isobutyrylcarnitine as a Biomarker of OCT1 Activity and Interspecies Differences in its Membrane Transport
Source: Front Pharmacol. 2021 May 10;12:674559. doi: 10.3389/fphar.2021.674559 (PMC8141810; doi:10.3389/fphar.2021.674559)

Figure S5

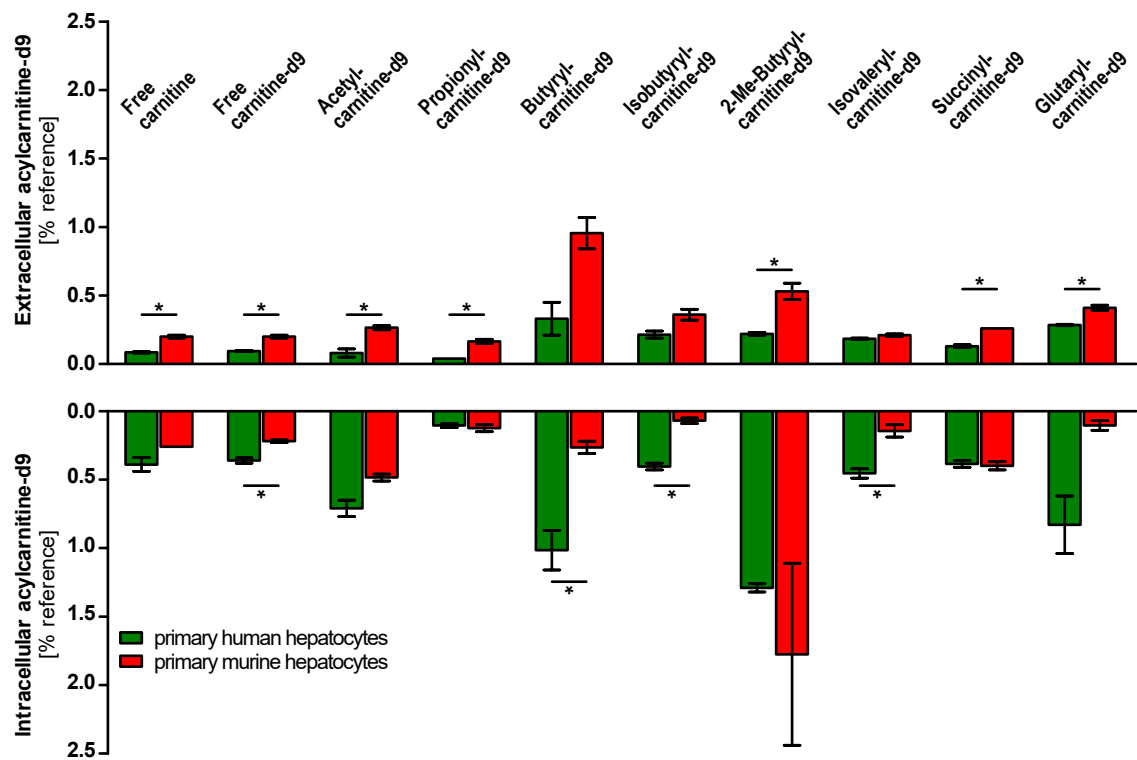

Supplement: Supplementary file 2 [file Image5.PDF]

Figure S6

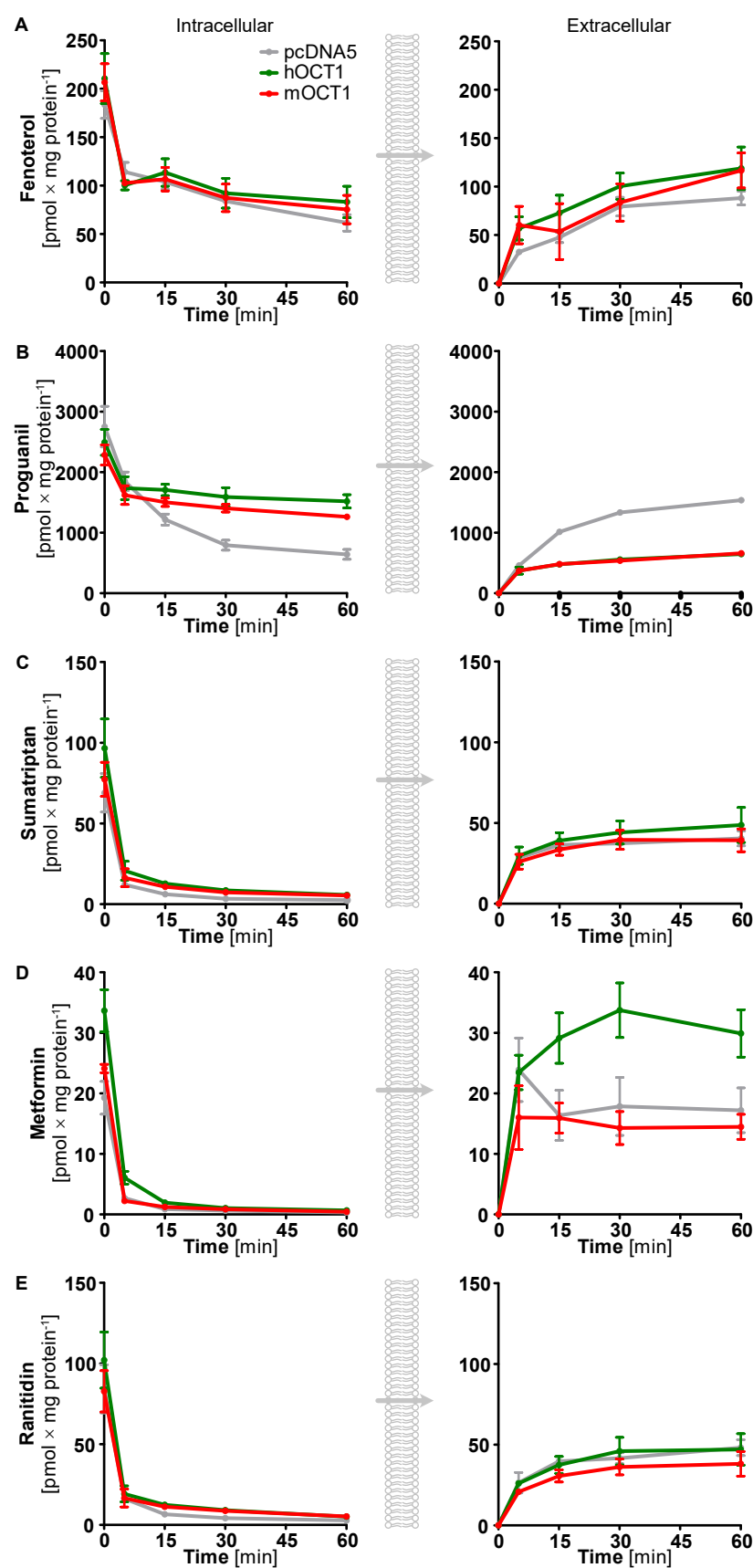

Supplement: Supplementary file 5 [file Image6.PDF]

Figure S4

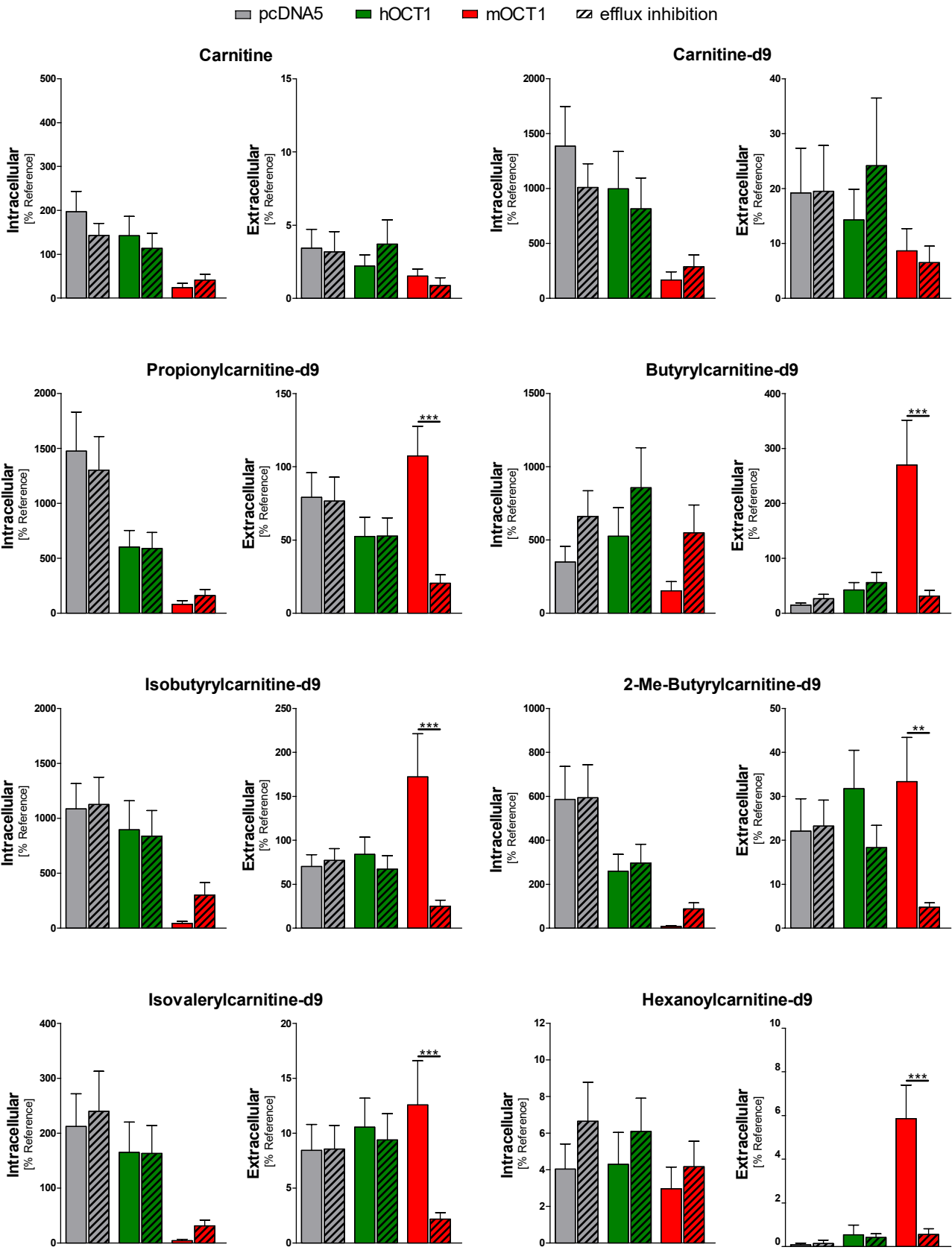

Supplement: Supplementary file 6 [file Image4.PDF]

Figure S2

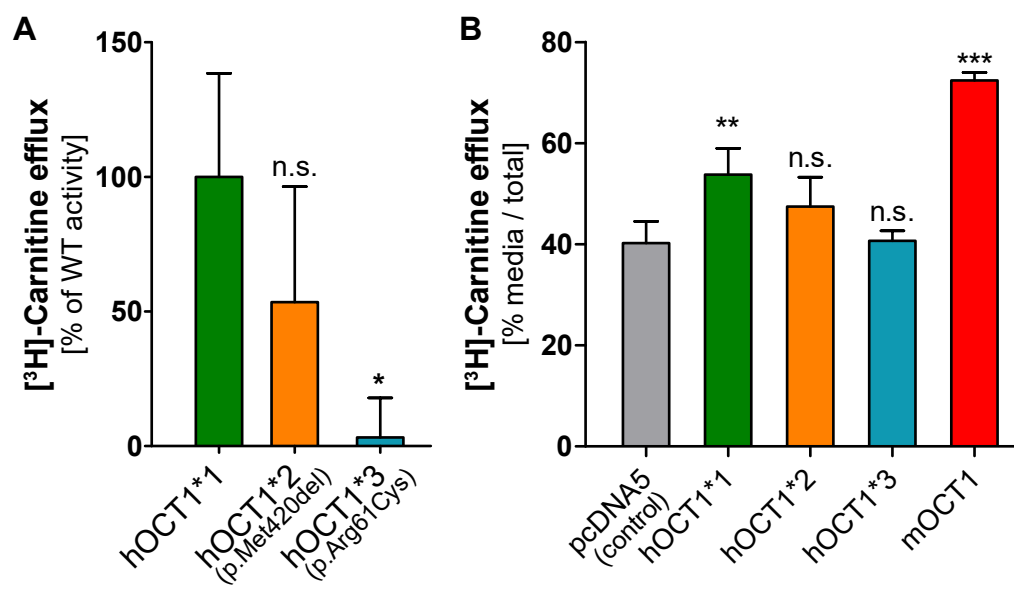

Supplement: Supplementary file 7 [file Image2.PDF]

Figure S3

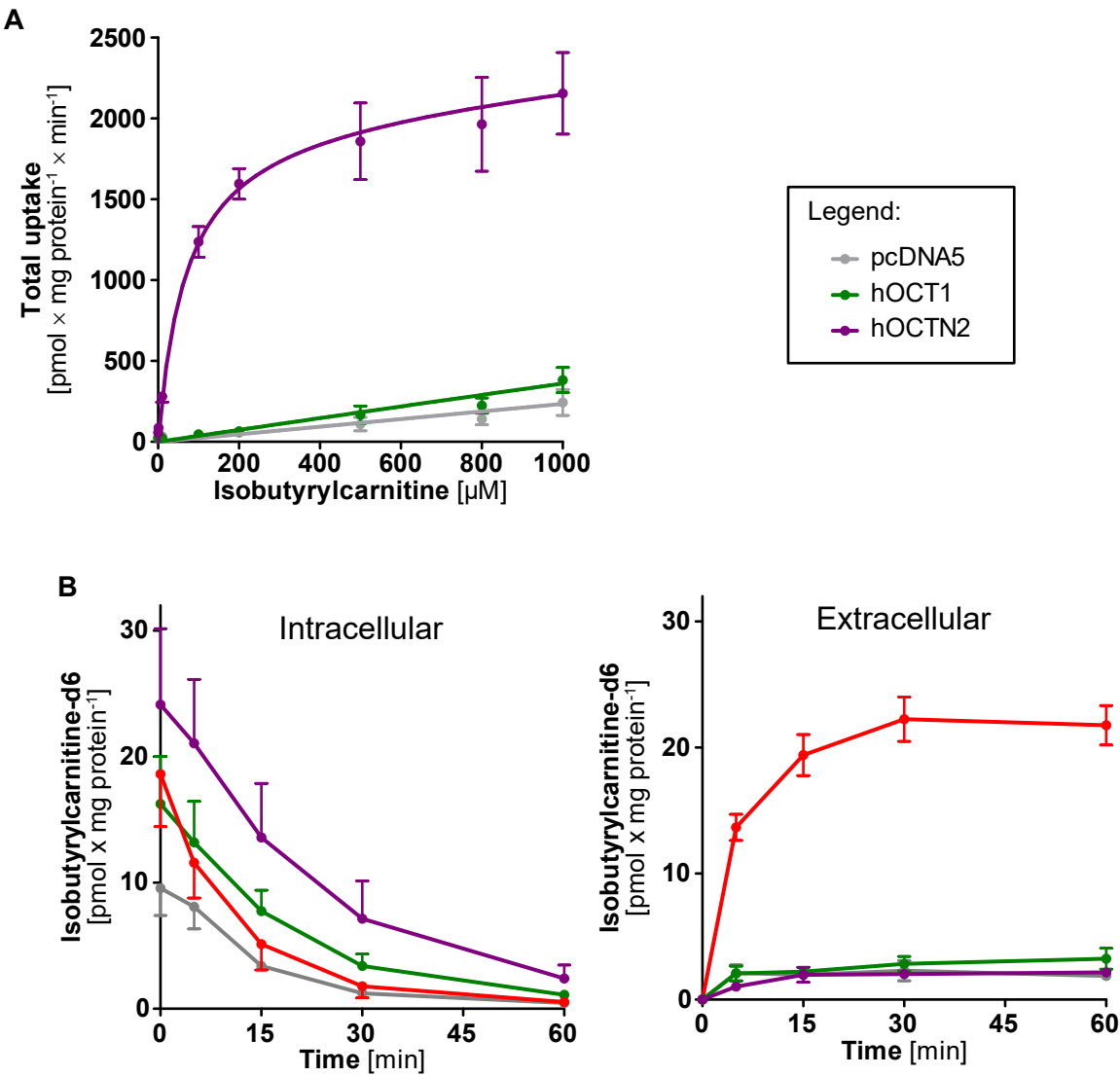

Supplement: Supplementary file 8 [file Image3.PDF]

Figure S1

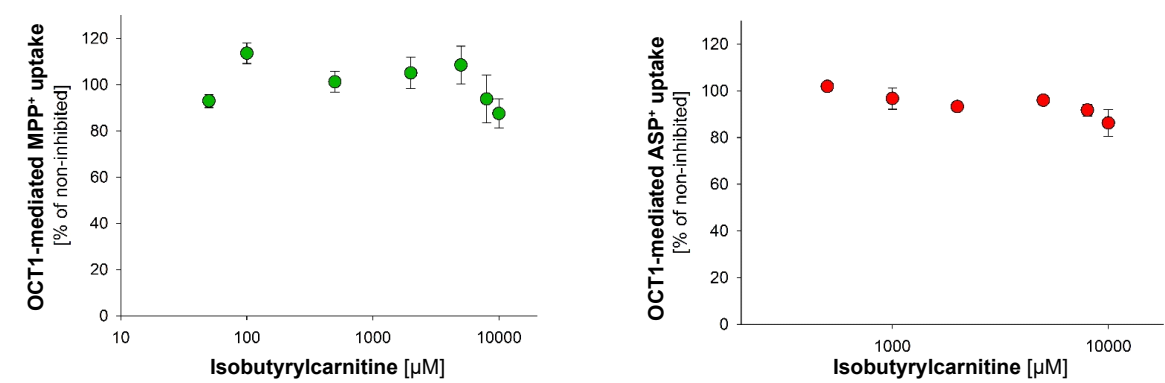

Supplement: Supplementary file 10 [file Image1.PDF]
