# Supplementary material for: Isobutyrylcarnitine as a Biomarker of OCT1 Activity and Interspecies Differences in its Membrane Transport
Source: Front Pharmacol. 2021 May 10;12:674559. doi: 10.3389/fphar.2021.674559 (PMC8141810; doi:10.3389/fphar.2021.674559)
Supplement: Supplementary file 3 [file Table1.PDF]

Table S1: Mass spectrometric parameters and selected chemical properties for acylcarnitine formation measurements as well as the efflux of known OCT1 substrates and IBC and valine uptake.

Compound	Structure	pk$_a$	Charge at pH 7.4	logD$_{7.4}$	RT (min)	Q1 (Da)	Q3 (Da)	DP (V)	CE (V)	CXP (V)
Carnitine	C0	4.20 (OH/O$^-$)	± (99.9%)	-4.12	2.7	162.2	85.0	60	29	16
Carnitine-d9						171.6				
Acetylcarnitine	C2	4.09 (OH/O$^-$)	± (100.0%)	-3.68	2.9	204.1	85.0	60	27	6
Acetylcarnitine-d9						211.5				
Propionylcarnitine	C3:0	4.19 (OH/O$^-$)	± (99.9%)	-2.98	3.6	218.2	85.0	60	28	6
Propionylcarnitine-d9						227.6				
Propionylcarnitine-d3					3.6	221.2	85.0	60	28	6
Butyrylcarnitine	C4:0	4.27 (OH/O$^-$)	± (99.9%)	-2.53	4.2	232.2	85.0	65	19	11
Butyrylcarnitine-d9						241.6				
Isobutyrylcarnitine	2-M-C3:0	4.27 (OH/O$^-$)	± (99.9%)	-2.43	4.1	232.1	85.0	70	25	15
Isobutyrylcarnitine-d6						238.1				
Isobutyrylcarnitine-d7						239.1				
Isobutyrylcarnitine-d9						241.6				
2-Me-butyrylcarnitine	2-M-C4:0	4.34 (OH/O$^-$)	± (99.9%)	-1.99	6.0	246.2	85.0	71	29	4
2-Me-butyrylcarnitine-d9						255.4				
Isovalerylcarnitine	3-M-C4:0	4.34 (OH/O$^-$)	± (99.9%)	-2.24	6.4	246.2	85.0	71	29	6
Isovalerylcarnitine-d9						255.4				
Succinylcarnitine	C4-DC	3.63 / 4.32 (OH/O$^-$)	- (99.9%)	-6.80	3.0	262.2	85.0	75	33	6
Succinylcarnitine-d9						271.4				
Hexanoylcarnitine	C6:0	4.22 (OH/O$^-$)	± (99.9%)	-1.64	14.7	260.2	85.0	76	31	6
Hexanoylcarnitine-d9						269.6				
Glutarylcarnitine	C5-DC	3.33 / 4.21 (OH/O$^-$)	- (99.9%)	-6.44	3.5	276.2	85.0	75	33	6
Glutarylcarnitine-d9						285.2				
Fenoterol					7.5	304.1	107.1	80	44	12
Fenoterol-d6					7.4	310.3	109.1	80	40	12
Metformin					2.9	130.0	71.0	40	35	10
Buformin					4.00	158.0	60.0	40	35	10
Proguanil					8.1	254.2	170.2	75	24	3
Proguanil-d6					7.9	260.3	170.2	75	24	10
Ranitidine					4.5	315.3	176.0	65	24	11
Ranitidine-d6					4.5	321.2	176.0	65	25	15
Sumatriptan					6.4	296.2	58.2	50	30	12
Sumatriptan-d6					6.4	302.2	64.2	70	30	12
Tyramine-d4					4.0	142.0	125.0	43	15	11
Valine					3.3	118.1	72.1	31	15	12
Valine-d8					3.3	126.1	80.0	39	17	14
Choline-d9					2.9	113.1	69.1	66	27	12

RT – retention time; Q1 – mass in first quadrupole; Q3 – mass in third quadrupole; DP – declustering potential; CE – collision energy; CXP – collision cell exit potential; Calculation and prediction of pk$_a$ values, charge and logD were performed with MarvinSketch (version 19.8, ChemAxon, Budapest, Hungary, www.chemaxon.com)
