# Supplementary material for: Isobutyrylcarnitine as a Biomarker of OCT1 Activity and Interspecies Differences in its Membrane Transport
Source: Front Pharmacol. 2021 May 10;12:674559. doi: 10.3389/fphar.2021.674559 (PMC8141810; doi:10.3389/fphar.2021.674559)
Supplement: Supplementary file 4 [file Table2.PDF]

Table S2: IBC plasma concentrations in healthy volunteers

Volunteer No.	OCT1 genotype	No of active OCT1 alleles	Plasma IBC (ng/mL) SD1	Plasma IBC (ng/mL) SD2	Plasma IBC (ng/mL) SD3	Plasma IBC (ng/mL) SD4	Plasma IBC (ng/mL) SD5	Plasma IBC (ng/mL) SD6	Mean plasma IBC (ng/mL)
1	H1/H1	2	32.3	32	28.2		38.8		32.8
2	H1/H4	1	7.6	14.7	13.8	12.5	14.8		12.7
3	H1/H2	1	12.0	10.4	14.9	16.2	17.4		14.2
4	H2/H3	0	12.0	10.2	11.6				11.3
5	H1/H1	2	16.8	43.6	31.4	19.5	24.2		27.1
6	H1/H1	2	22.5						22.5
7	H1/H1	2	16.8	19.6		27.2	5.99		17.4
8	H1/H1	2	15.7	8.02	12.2	14.8		10.3	12.2
9	H1/H4	1	6.5						6.5
10	H1/H3	1	16.3						16.3
11	H1/H1	2	8.8	13.8	11.5				11.4
12	H2/H3	0	8.6	9.58	10.1	6.5	10.8		9.1
13	H1/H1	2	25.3	20	22.6	21.8	29.8		23.9
14	H1/H1	2	14.8	12.2	48.5	20.5			24.0
15	H2/H2	0	7.3	9.83	6.58	5.94	6.88	12.1	8.1
16	H1/H1	2	19.9	25.9	16.6	22.5	14.8	23.7	20.6
17	H1/H2	1	9.1	10.5	12.3	8.55			10.1
18	H1/H1	2	15.6	16.2	7.88	7.94	19.4	14.3	13.6
19	H1/H3	1	12.8	21.5	11.8	32.9	10.9		18.0
20	H2/H2	0	13.1	7.54	6.3				9.0
21	H1/H3	1	9.4	15.9	16.6	17.5	21.9		16.3
22	H2/H2	0	3.6	7.1	5.87	8.08	10.3	8.77	7.3
23	H2/H3	0	8.1	14.5	9.65	10.2			10.6
24	H1/H1	2	9.2	9.38	15.2	9.82	21.4	15.6	13.4
25	H1/H1	2	49.9	54.5	54.5	28.5	66.7		50.8
26	H1/H1	2	22.2						22.2
27	H1/H1	2					15.5		15.5
28	H1/H2	1	10.6	22.9	9.35	10.4	8.95		12.4
29	H3/H3	0	4.7	4.99	5.41	7.35	5.75		5.6
30	H1/H2	1	10.6	9.92	12.1		19.2		13.0
40	H1/H2	1					9.82		9.8
41	H3/H3	0	3.6	4.55	4.1		4.02	4.11	4.1
42	H3/H5	0	7.9						7.9
43	H2/H3	0	6.2	5.4	6.06	6.3	7.83		6.4
44	H1/H1	2	31.6	33.2	19.1		27.5		27.9
45	H3/H4	0	4.8	3.26	4.39	4.18	3.15	2.39	3.7
46	H1/H4	1	7.5						7.5
47	H2/H3	0	4.3	4.94	4.38	6.94	2.58		4.6
48	H1/H4	1	8.8	5.51	13	13.7	8.85		10.0
49	H1/H4	1	16.2	10.5	27		15.5		17.3
50	H1/H3	1	9.73	10.9	10.3	9.01	11.3		10.2
51	H1/H1	2		16.9	15.1	12.5	14.5		14.8
52	H1/H4	1			30.4		18.2		24.3

Volun-teer No.	OCT1 geno-type	No of active OCT1 alleles	Plasma IBC (ng/mL) SD1	Plasma IBC (ng/mL) SD2	Plasma IBC (ng/mL) SD3	Plasma IBC (ng/mL) SD4	Plasma IBC (ng/mL) SD5	Plasma IBC (ng/mL) SD6	Mean plasma IBC (ng/mL)
53	H1/H1	2		17.1		15.6	12.2	20.1	16.3
54	H3/H3	0	3.9	5.58	3.95	6.37	9.24		5.8
55	H1/H1	2				14.9			14.9
56	H1/H4	1	16.2						16.2
57	H2/H4	0		7.37			7.53		7.5
58	H2/H2	0		6.16	8.03	9.03	12.8		9.0
59	H2/H2	0			10.6	13.9			12.3
60	H1/H3	1	30.5		9.22		8.86		16.2
61	H3/H3	0	3.6						3.6
62	H1/H3	1			18.2				18.2
63	H1/H1	2					22.8	22.2	22.5
64	H1/H1	2			7.65		8.24	31.4	15.8
65	H1/H1	2				13.9		21.5	17.7
66	H2/H5	0				4.96		7.27	6.1
67	H1/H3	1		23.4					23.4
68	H3/H3	0	5.6			6.13	4.38	8.06	6.0
69	H1/H1	2						62.1	62.1
70	H2/H3	0						16	16.0
71	H3/H5	0	3.1					7.14	5.1
72	H3/H3	0	4.4						4.4
73	H1/H2	1					6.48		6.5
74	H1/H3	1				11.9			11.9

IBC – isobutyrylcarnitine; SD – study day; H – haplotype
