# Supplementary material for: Isobutyrylcarnitine as a Biomarker of OCT1 Activity and Interspecies Differences in its Membrane Transport
Source: Front Pharmacol. 2021 May 10;12:674559. doi: 10.3389/fphar.2021.674559 (PMC8141810; doi:10.3389/fphar.2021.674559)
Supplement: Supplementary file 9 [file DataSheet1.PDF]

Supporting Information

Isobutyrylcarnitine as biomarker of OCT1 activity and interspecies differences in its membrane transport

Short title: Isobutyrylcarnitine as an OCT1 biomarker

Ole Jensen[1], Johannes Matthaei[1], Henry G. Klemp[2], Marleen J. Meyer[3], Jürgen Brockmöller[1], and Mladen V. Tzvetkov[3]

[1] Department of Clinical Pharmacology, University Medical Center Göttingen, D-37075 Göttingen, Germany

[2] Department of Pediatrics and Adolescent Medicine, University Medical Center Göttingen, D-37075 Göttingen, Germany

[3] Institute of Pharmacology, Center of Drug Absorption and Transport (C_DAT), University Medicine Greifswald, D-17489 Greifswald, Germany

Supporting Materials and Methods

Isobutyrylcarnitine blood and urine concentration analyses

Peripheral venous blood samples, anticoagulated with ethylenediaminetetraacetic acid (EDTA), were centrifuged within 30 minutes after withdrawal (3,100 × g, 10 minutes, room temperature) and plasma was immediately stored at -20 °C. The plasma concentrations of IBC were analyzed by liquid chromatography-coupled tandem mass spectrometry (LC-MS/MS) as follows: Plasma samples were mixed with twice the volume precipitation reagent (10% (v/v) methanol, 90% (v/v) acetonitrile, including the internal standard IBC-d6 (Santa Cruz Biotechnology, Heidelberg, Germany)) and shaken for 15 minutes on a rotation shaker. After centrifugation (13,000 rpm, 15 minutes, room temperature), two thirds of the supernatant were transferred to a fresh reaction tube and evaporated at 40 °C under nitrogen flow. The residue was reconstituted under shaking (VXR Vibrax; IKA Werke, Staufen, Germany) in 0.1% formic acid and briefly spun down before separation and quantification by HPLC-MS/MS. Isobutyrylcarnitine and other acylcarnitines were measured together with creatinine in urine samples after precipitation (3,000 × g for 30 minutes) and dilution (1:51 in 0.1 % formic acid). The *Shimadzu Nexera* HPLC system included a *LC-30AD* pump, a *SIL-30AC* autosampler, a *CTO-20AC* column oven, and a *CBM-20A* controller (all Shimadzu, Kyoto, Japan) and was used to separate 10 µl injected sample on a *Brownlee SPP RP-Amide* column (4.6 x 100 mm inner dimensions, 2.7 µm particle size; Perkin Elmer, Rodgau, Germany) with a *Phenomenex C18* pre-column (4 x 2 mm, Phenomenex, Aschaffenburg, Germany) prior to detection with an *API 4000* tandem mass spectrometer (AB Sciex, Darmstadt, Germany). The mobile phase consisted of 0.1% (v/v) formic acid, 0.43% (v/v) methanol, and 2.57% (v/v) acetonitrile. IBC and the

internal standard IBC-d6 were quantified using the mass transitions and voltages listed in Table S1.

Uptake of isobutyrylcarnitine or valine

Transport experiments for cellular uptake of IBC or valine were performed with HEK293 cells stably transfected to overexpress hOCT1 or mOCT1. As a control, cells transfected with the empty vector pcDNA5 were used. The generation and validation of the cell lines was described elsewhere (1, 2). Transport experiments were initiated by plating of 6 × 10^5 HEK293 cells per well in 12-well plates pre-coated with poly-D-lysine (Sigma-Aldrich, Taufkirchen, Germany) 48 hours ahead of the actual experiment to reach confluence. To determine IBC or valine uptake in cells cultured in a valine-containing medium, depletion of excessive intracellular IBC or valine was achieved by incubation of the cells in HBSS+ for 30 minutes prior to uptake experiments. Cells were washed once with 2 ml 37 °C HBSS (pH 7.4, supplemented with 10 mM HEPES – hereafter referred to as HBSS+) and incubated with increasing concentrations of IBC or valine in 37 °C HBSS+. After two minutes, the reaction was stopped by the addition of 2 ml ice-cold HBSS+. Next, cells were washed twice with 2 ml ice-cold HBSS+ and lysed in 500 µl 80% acetonitrile (LGC Standards, Wesel, Germany) supplemented with internal standard (IBC – tyramine-d4 (Santa Cruz Biotechnology, Heidelberg, Germany); valine – choline-d9 (Sigma-Aldrich, Taufkirchen, Germany). Accumulation of intracellular IBC or valine was quantified by HPLC-MS/MS.

Efflux of ^{3}H-carnitine, isobutyrylcarnitine-d6, and known substrates

Efflux of radiolabeled carnitine was performed based on the description of Kim and colleagues (3). Six hundred thousand cells were seeded per well in a 12-well plate pre-coated with poly-D-lysine 48 hours ahead of the transport experiment to reach

confluence. For the experiment, cells were washed once with 37 °C PBS supplemented with 20 mM HEPES pH 7.4 (from now on called PBS^+). Next, cells were incubated in 500 µl uptake medium (PBS^+ supplemented with a final concentration of 20 µM carnitine and 0.1 % (v/v) ^{3}H-carnitine 1 µCi/µl) for 30 minutes and washed rapidly three times with PBS^+. Five hundred µl efflux medium (PBS^+ supplemented with 20 µM carnitine) were added to collect the exported ^{3}H-carnitine and the supernatant was removed after different time points. Cells were then washed twice with ice-cold PBS^+ and lysed with 0.1 N NaOH solution containing 0.1 % SDS. Remaining intracellular content as well as extracellular accumulation was determined in a Beckman scintillation system LS 6500 (Beckman Coulter, Krefeld, Germany) by addition of 8 ml Aquasafe 500+ liquid scintillator (Zinsser Analytics, Frankfurt am Main, Germany) to 400 µl sample.

Experiments with IBC were performed by analogy to the above-mentioned conduction. Instead of radiolabeled carnitine, 20 µM IBC-d6 (Toronto Research Chemicals, North York, Canada) was used in the uptake medium, and HBSS was used instead of PBS throughout the experimental procedure. The efflux buffer in experiments adjusted for intracellular accumulation did not contain 20 µM carnitine as described above for experiments with radiolabeled carnitine. The lysis of cells after washing was accomplished with 80% acetonitrile with 10 ng/ml tyramine-d4 as internal standard.

Experiments to test for possible efflux activity of OCT1 with other substrates were conducted analogously to efflux experiments with IBC-d6. However, to adjust the intracellular accumulation after the 30 minutes incubation period, extracellular concentrations of fenoterol, metformin, proguanil, ranitidine, and sumatriptan (all Sigma-Aldrich, Taufkirchen, Germany) were adjusted to the uptake of the control cell line, which was incubated with 20 µM of each substance. Lysis of cells was accomplished with 80% acetonitrile with the appropriate internal standard for each of

the substances individually (fenoterol-d6 – Biozol, Eching, Germany; buformin – Wako Chemicals, Neuss, Germany; proguanil-d6 – TRC, Toronto, Canada; ranitidine-d6 – TRC, Toronto, Canada; sumatriptan-d6 – Santa Cruz Biotechnology, Heidelberg, Germany).

Efflux of d9-acylcarnitines

Formation of acylcarnitines was investigated by the addition of carnitine-d9 to the cell medium. Uptake and subsequent coupling of acyl residues to the deuterated carnitine led to heavy acylcarnitines, which were analyzed by LC-MS/MS. The experiment was conducted as follows: Six hundred thousand cells were seeded per well in a 12-well plate pre-coated with poly-D-lysine 48 hours ahead of the transport experiment to reach confluence. Twenty-four hours prior to the experiment, the medium was replaced by DMEM containing 20 µM carnitine-d9 (Sigma-Aldrich, Taufkirchen, Germany). Alternatively, cells were incubated with 20 µM carnitine-d9 in HBSS+ for 30 minutes. After carnitine-d9 incubation, cells were washed with 1 ml 37 °C HBSS+ and incubated in 500 µl efflux medium (HBSS+) for 30 minutes. After this, the supernatant was collected and cells were washed twice with 1 ml ice-cold HBSS+ before lysis with 500 µl 80% acetonitrile supplemented with 10 ng/µl propionylcarnitine-d3 as internal standard. Intracellular as well as extracellular content of d9-labeled acylcarnitine species were quantified using HPLC-MS/MS. For this, workup of cell lysates and the precipitation of substances in the supernatant was conducted as described above. Substances with identical mass transitions were separated by HPLC in the best possible way. Due to the lack of pure d9-labeled acylcarnitines, efflux was not measured quantitatively but normalized to the intracellular carnitine concentration in the control cell line.

Formation of isobutyrylcarnitine-d7 from valine-d8

The valine-dependent formation of IBC was investigated by incubating HEK293 cells overexpressing mouse or human OCT1 with deuterated valine (valine-d8). Cells were plated in 12-well plates as described above. Prior to incubation with 20 µM valine-d8 in HBSS+, cells were washed once with 37 °C HBSS+. After predefined intervals, the supernatant medium was removed and collected. Cell were then washed twice with 1 mL ice-cold HBSS+ and 500 µL lysis buffer including the internal standard was added to each well. Workup of cell lysates and the precipitation of substances in the supernatant was conducted as described above. IBC-d7 was then measured via HPLC-MS/MS as described below. Due to the lack of pure IBC-d7, the analysis was not performed quantitatively but normalized to uptake into control cells after 30 minutes.

Efflux of acylcarnitines by murine and human hepatocytes

Efflux of deuterated acylcarnitines was comparatively examined with mouse and human cryopreserved hepatocytes (Life Technologies, Darmstadt, Germany). Mouse hepatocytes comprised a pool from several animals, while human hepatocytes derived from a single donor with two fully active *OCT1* alleles (homozygous *OCT1*1* genotype). The cryopreserved hepatocytes were rapidly thawed in a 37 °C water bath and poured into 47 ml of 37 °C cryopreserved hepatocyte recovery medium (*CHRM*, Life Technologies, Darmstadt, Germany) and pelleted by centrifugation (100 × g, 10 min, 4 °C). The cell pellet was resuspended in cryopreserved hepatocyte plating medium (*CHPM*, Life Technologies, Darmstadt, Germany) supplemented with 20 µM carnitine-d9 (Sigma-Aldrich, Darmstadt, Germany) and 3 × 10^5 cells were seeded per well of a 24-well plate coated with collagen-I (Life Technologies, Darmstadt, Germany). Hepatocytes were incubated for 4 hours at 5% CO_2 and 37 °C to allow attachment of

the cells. After this, efflux of d9-labeled acylcarnitines was performed as described above and quantified as described below.

Protein measurement

In vitro uptake experiments in HEK293 cells overexpressing transporters of interest were normalized by total protein. In every experiment, proteins were measured in representative wells by using the bicinchoninic acid assay(4). Protein measurement was performed in samples after lysis in RIPA buffer (in uptake and efflux experiments with deuterated carnitine and acylcarnitines, valine, fenoterol, metformin, proguanil, ranitidine, sumatriptan) or 0.1 N HCl solution (radiolabeled carnitine).

HPLC-MS/MS measurement of deuterated acylcarnitine species and known OCT1 substrates

Intracellular and extracellular concentrations of deuterated and non-labeled acylcarnitines and carnitine were quantified by HPLC with tandem-mass spectrometric detection. The *Shimadzu Nexera* HPLC system consisted of a *LC-30AD* pump, a *SIL-30AC* autosampler, a *CTO-20AC* column oven and a *CBM-20A* controller (Shimadzu, Kyoto, Japan). Separation was achieved with a *Brownlee SPP RP-Amide* column (100 x 4 mm inner dimensions, 2.7 µm particle size; Perkin Elmer) with a *Phenomenex C18* pre-column (2x4 mm, Phenomenex, Aschaffenburg, Germany) and 40 °C oven temperature. The mobile phase for separation of acylcarnitine species, fenoterol, ranitidine, and sumatriptan consisted of 0.1% (v/v) formic acid, 6.86% (v/v) acetonitrile, and 1.14% (v/v) methanol. Elution of valine and metformin was achieved isocraticly with a mobile phase of 0.1% (v/v) formic acid, 2.57% (v/v) acetonitrile, and 0.43% (v/v) methanol. Proguanil was quantified after elution with a mobile phase of 0.1% (v/v) formic acid, 17.14% (v/v) acetonitrile, and 2.86% (v/v) methanol. Detection of the

investigated substances and appropriate internal standards was performed with an *API 4000* tandem mass spectrometer (AB SCIEX, Darmstadt, Germany) with parameters listed in Table S1.

Calculating active transport and efflux

Active transport was calculated as the difference between uptake into cells overexpressing a transporter and the control cell line. The Michaelis-Menten constant K_m and the maximum velocity of transport v_{max} were estimated using regression analysis with the Michaelis-Menten equation for each experiment individually. Means and standard errors of the means were calculated from K_m and v_{max} values of three or more independent experiments. Therefore, data are expressed as mean ± standard error of the mean (mean ± SEM).

If not given in absolute values, efflux was calculated as the ratio of extracellularly accumulated substance over the sum of extracellular and intracellular (total) substance. Inversely, the percentage of intracellular substance left was calculated as the intracellular amount divided by the total quantity of the substance.

Metabolomics

To study endogenous substrates of mouse and human OCT1, we performed untargeted metabolomics. Cells were plated in 12-well plates, pre-coated as described above, washed once with HBSS+ and incubated with 400 µl pooled fresh frozen plasma from three healthy humans for 5 minutes. The supernatant plasma was immediately frozen in liquid nitrogen. Cells were washed with ice-cold HBSS+ and mechanically detached in 500 µl ice-cold phosphate buffer (10 mM, pH 7.5) and frozen in liquid nitrogen. Cells were lysed using three freeze-thaw cycles and normalized by total protein concentration (after bicinchoninic acid assay). To remove lipids and

proteins, a modified Bligh and Dyer method (5) was used as follows: 500 µl sample were extracted with 1000 µl methanol/HCl/corticosterone-d8 (premixed of 1000 µl methanol, 100 µl 3 M HCl and 1 µl corticosterone-d8 (Toronto Research Chemicals, Toronto, Canada)). The remaining protein pellet was then subjected to liquid-liquid extraction with 500 µl chloroform, reunited with the liquid extract to extract further from the supernatant. The aqueous phase, containing the hydrophilic metabolites, was subjected to a zwitterionic HILIC solid-phase extraction (ZIC-HILIC SPE column (Macherey-Nagel, Düren, Germany). For this, the aqueous phase was mixed with 1.5 ml HILIC-SPE buffer (70 % (v/v) tetrahydrofuran / 25 % (v/v) acetonitrile / 5 % (v/v) methanol). Column initiation was performed with 2 ml ddH_2O and equilibrated with 2 ml HILIC buffer. The sample was loaded onto the column, incubated for 15 minutes and unbound sample removed. Sample elution was performed by addition of 2.5 ml ddH_2O after incubation for 15 minutes. The eluate was dried by vacuum centrifugation. The dried residues were dissolved in 100 µl HPLC-phase B (90 % acetonitrile / 10 % 30 mM ammonium acetate in ddH_2O, pH 7.0) and transferred to LC-MS vials (Waters, Eschborn, Germany). A quality control of all samples from one matrix (cell lysate and supernatant/plasma separately) was prepared by mixing 10 µl of each sample. Separation of samples was performed on a Waters I-Class UPLC with a 250 mm × 2.1mm, 3.5 µm *Sequant ZIC-HILIC* column (Merck, Darmstadt, Germany), with mobile phase A (10% Acetonitrile, 90% 30 mM ammonium acetate in ddH_2O, pH 7.0) and mobile phase B (see above). The separation used a flow of 0.2 ml/min, starting with initially 90% mobile phase B for 2 min, a linear gradient to 10% B for 18 min, a linear gradient to 90% B for 5 min and column equilibration for 5 min prior to next injection. Five µl sample were injected, with measurement of quality control samples every 10-15 samples.

Detection of metabolites was performed using a *Xevo G2-S QToF* mass spectrometer (Waters, Düren, Germany). ESI capillary voltage was set to 1.5 kV and 2.5 kV in negative and positive mode respectively, cone voltage was 50 V, source offset 60 V, source temperature set to 120°C and desolvation temperature set to 500°C. Analysis was performed using *MassLynx 4.1* (Waters, Milford, US), *Progenesis QI 2.4* (Nonlinear Dynamics, Newcastle upon Tyne, UK) as well as *Metaboanalyst 4.0* (6). Data for heat map generation were normalized using pareto-scaling and log-transform, and lowest ANOVA p-values per features were used for prefiltering. Clustering used Ward-Algorithm and Euclidean distance. Identification was achieved by an in-house database of specific retention times and masses of approx. 400 metabolites, as well as the HMDB database via *Progenesis* software (7).

PPARα activation

For isobutyrylcarnitine-d9 measurements after PPARα activation, 600,000 cells were plated in 12-well plates as described above. After 24 hours, the DMEM was replaced by DMEM containing only 20 µM carnitine-d9 or 20 µM carnitine-d9 and 33 µM fenofibrate or 33 µM of the PPARα antagonist MK886. After another 24 hours, medium was removed and cells were washed twice with 4 °C HBSS+ and lysed with 80% acetonitrile and propionylcarnitine-d3 as internal standard. Sample preparation and LC-MS/MS measurement was performed as described above.

References

(1) Saadatmand, A.R., Tadjerpisheh, S., Brockmöller, J. & Tzvetkov, M.V. The prototypic pharmacogenetic drug debrisoquine is a substrate of the genetically polymorphic organic cation transporter OCT1. *Biochem Pharmacol* **83**, 1427-1434 (2012).

(2) Meyer, M.J. *et al.* Differences in metformin and thiamine uptake between human and mouse organic cation transporter OCT1: structural determinants and potential consequences for intrahepatic concentrations. *Drug Metabolism and Disposition* 10.1124/dmd.120.000170, DMD-AR-2020-000170 (2020).

(3) Kim, H.I. *et al.* Fine Mapping and Functional Analysis Reveal a Role of SLC22A1 in Acylcarnitine Transport. *The American Journal of Human Genetics* 10.1016/j.ajhg.2017.08.008, (2017).

(4) Smith, P.K. *et al.* Measurement of protein using bicinchoninic acid. *Analytical Biochemistry* **150**, 76–85 (1985).

(5) Bligh, E.G. & Dyer, W.J. A rapid method of total lipid extraction and purification. *Canadian journal of biochemistry and physiology* **37**, 911-917 (1959).

(6) Chong, J., Wishart, D.S. & Xia, J. Using MetaboAnalyst 4.0 for Comprehensive and Integrative Metabolomics Data Analysis. *Current Protocols in Bioinformatics* **68**, e86 (2019).

(7) Wishart, D.S. *et al.* HMDB: the Human Metabolome Database. *Nucleic Acids Research* **35**, D521-D526 (2007).
